# Supplementary material for: CD206+ macrophages facilitate wound healing through interactions with Gpnmbhi fibroblasts
Source: EMBO Rep. 2025 Jun 10;26(14):3679–704. doi: 10.1038/s44319-025-00496-4 (PMC12287335; doi:10.1038/s44319-025-00496-4)
Supplement: Supplementary file 1 — Table EV1 [file 44319_2025_496_MOESM1_ESM.pdf]

Gpnmb^{hi} fibroblast

Upregulated gene sets

Gene set	NES	FDR q-val
Cholesterol Homeostasis	1.658	0.017
Fatty Acid Metabolism	1.672	0.021
mTORC1 Signaling	1.691	0.033

Downregulated gene sets

Gene set	NES	FDR q-val
KRAS Signaling DN	-1.696	0.071
Coagulation	-1.725	0.122

Plac8^{hi} fibroblast

Upregulated gene sets

Gene set	NES	FDR q-val
mTORC1 Signaling	1.825	0.016
Cholesterol Homeostasis	1.698	0.040
G2M Checkpoint	1.595	0.057
E2F Targets	1.550	0.073

Downregulated gene sets

Gene set	NES	FDR q-val
P53 Pathway	-1.625	0.106
KRAS Signaling DN	-1.686	0.142

Crabp1^{hi} fibroblast

Upregulated gene sets

Gene set	NES	FDR q-val
Cholesterol Homeostasis	1.749	0.001
mTORC1 Signaling	1.673	0.006
Peroxisome	1.621	0.011
MYC targets	1.527	0.033

Downregulated gene sets

Gene set	NES	FDR q-val
KRAS signaling DN	-2.464	0.004
Coagulation	-2.108	0.034
TNFα Signaling via NFκB	-1.318	0.147

Table EV1
GSEA of differentially regulated genes by CD206+ depletion

Molecular Signatures Database (MSigDB) hallmark gene sets that were positively and negatively enriched in Gpnmb^{hi}, Plac8^{hi} and, Crabp1^{hi} fibroblast subpopulations in Mrc1-DTR wounds compared to the control wounds (FDR < 0.15). NES, normalized enrichment score.
