# Supplementary figures and images for: CD206+ macrophages facilitate wound healing through interactions with Gpnmbhi fibroblasts
Source: EMBO Rep. 2025 Jun 10;26(14):3679–704. doi: 10.1038/s44319-025-00496-4 (PMC12287335; doi:10.1038/s44319-025-00496-4)

**Datasets EV 1**

**Top 10 differentially expressed genes in each cluster**

**
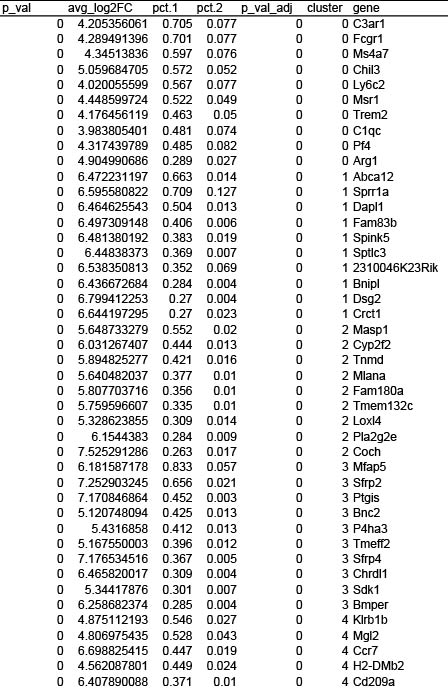
**

**
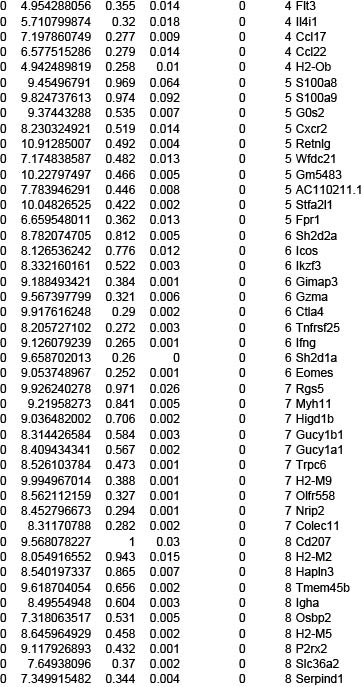

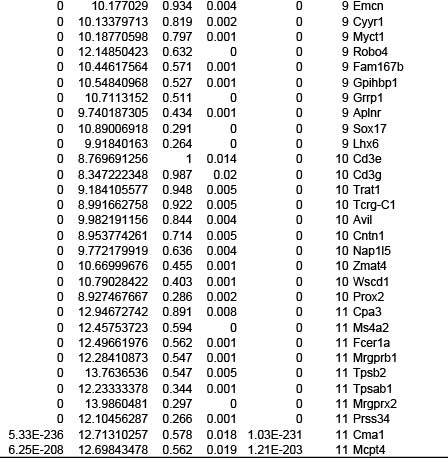
**

Supplement: Supplementary file 3 — Data Set EV1 [file 44319_2025_496_MOESM3_ESM.docx]

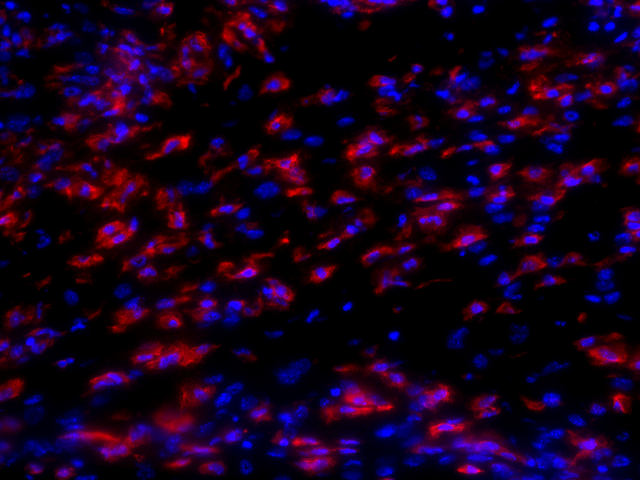

Supplement: Supplementary file 5 — Source data Fig. 1 [file 44319_2025_496_MOESM5_ESM.zip › Figure 1/1D/C1_401.tif]

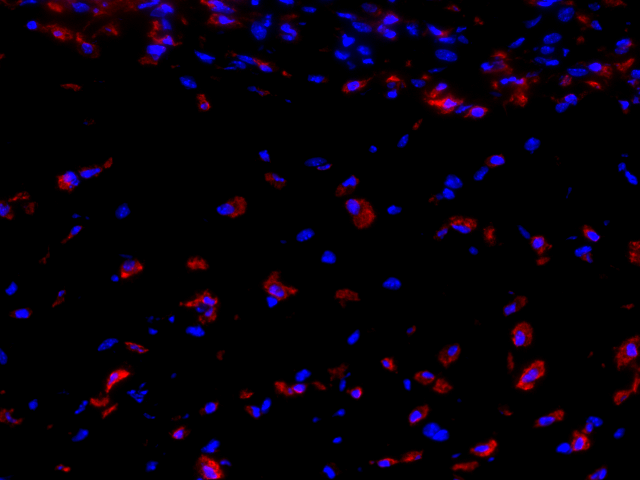

Supplement: Supplementary file 5 — Source data Fig. 1 [file 44319_2025_496_MOESM5_ESM.zip › Figure 1/1D/D2_401.tif]

## Slide 1
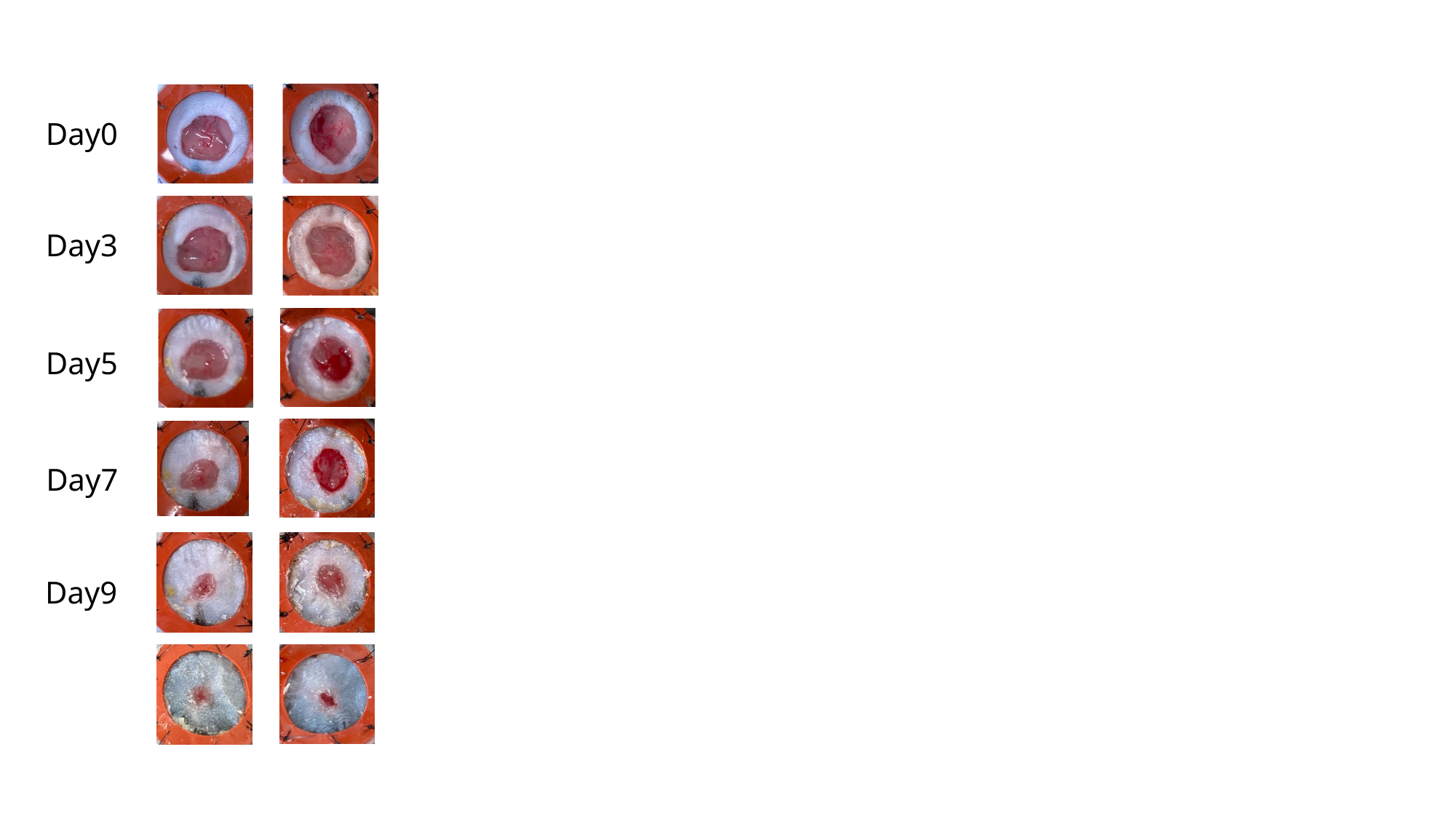

Day0
Day3
Day5
Day7
Day9

Supplement: Supplementary file 5 — Source data Fig. 1 [file 44319_2025_496_MOESM5_ESM.zip › Figure 1/1F/Mouse_skin_image.pptx]

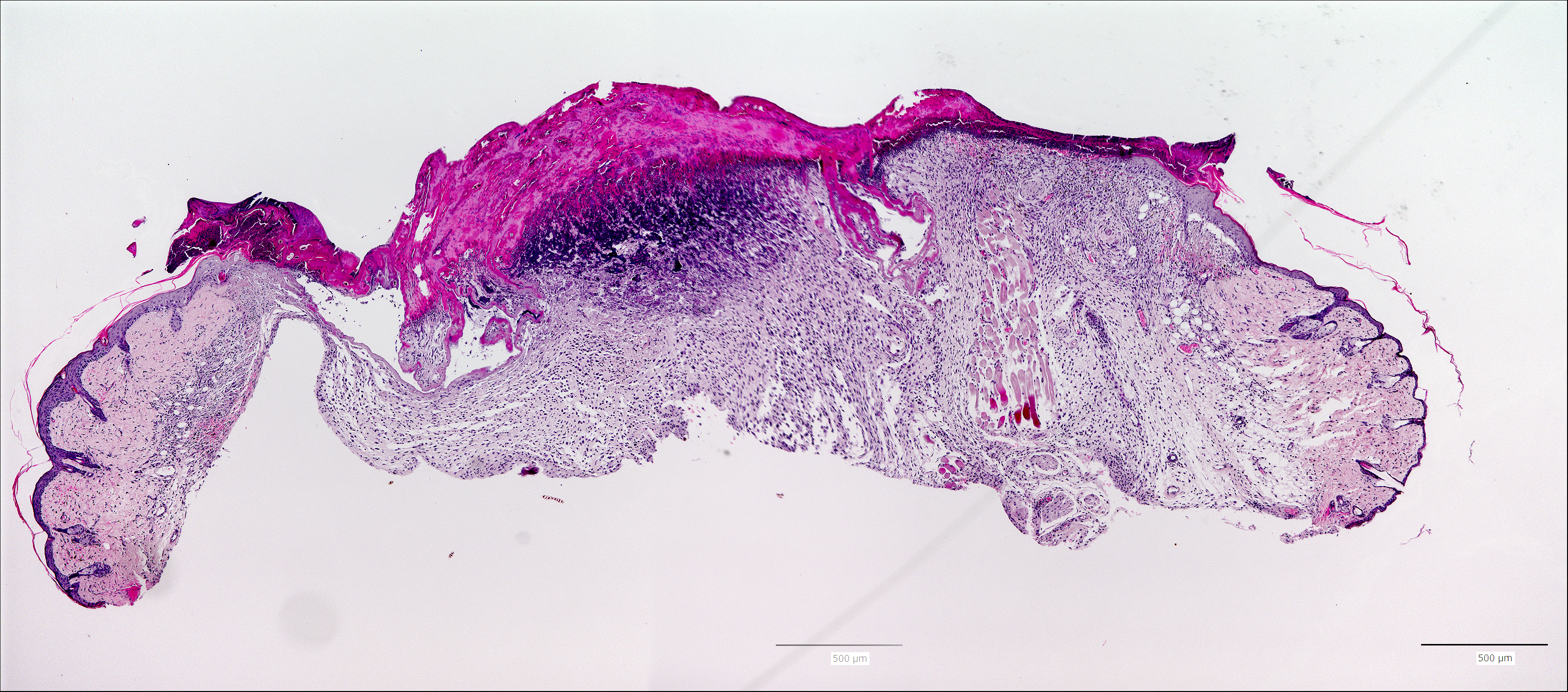

Supplement: Supplementary file 5 — Source data Fig. 1 [file 44319_2025_496_MOESM5_ESM.zip › Figure 1/1H/Day5 Control x4.tif]

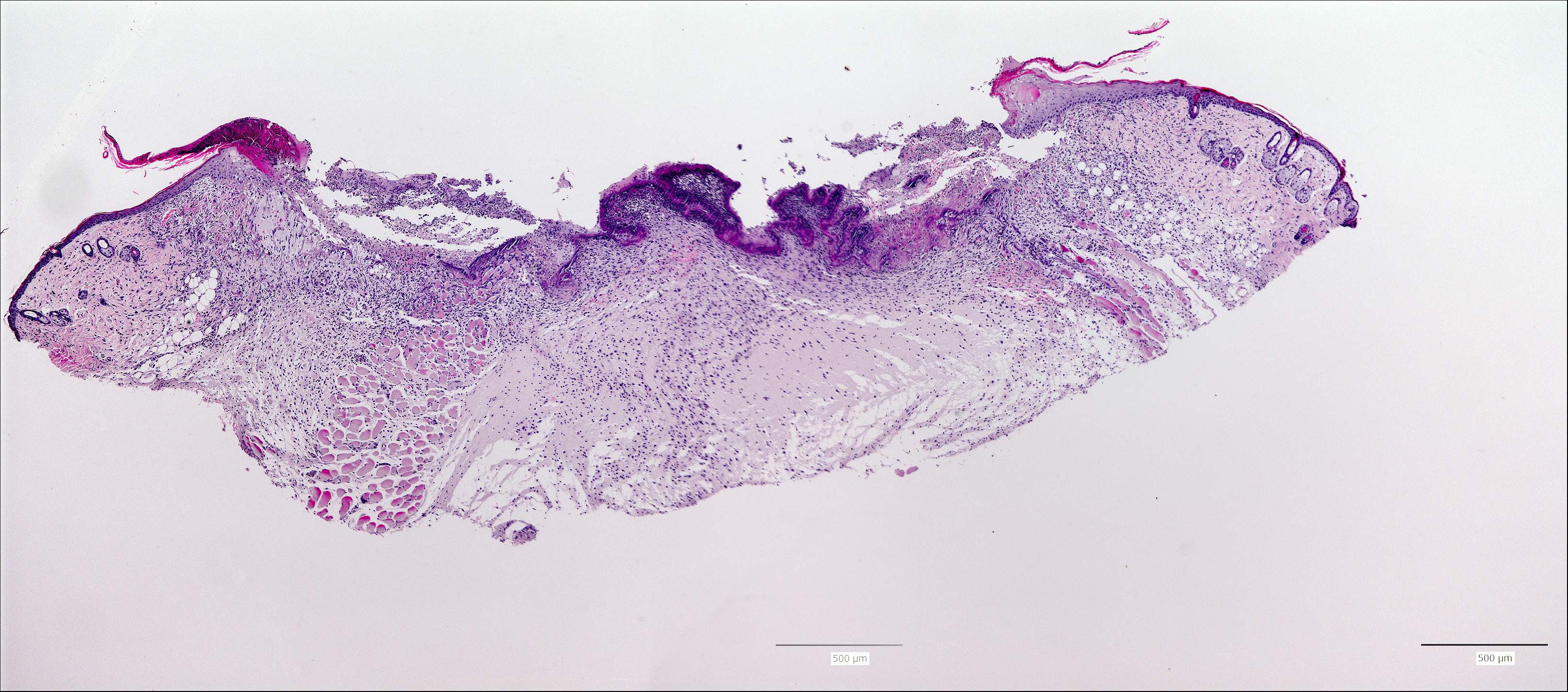

Supplement: Supplementary file 5 — Source data Fig. 1 [file 44319_2025_496_MOESM5_ESM.zip › Figure 1/1H/Day5 KO x4.tif]

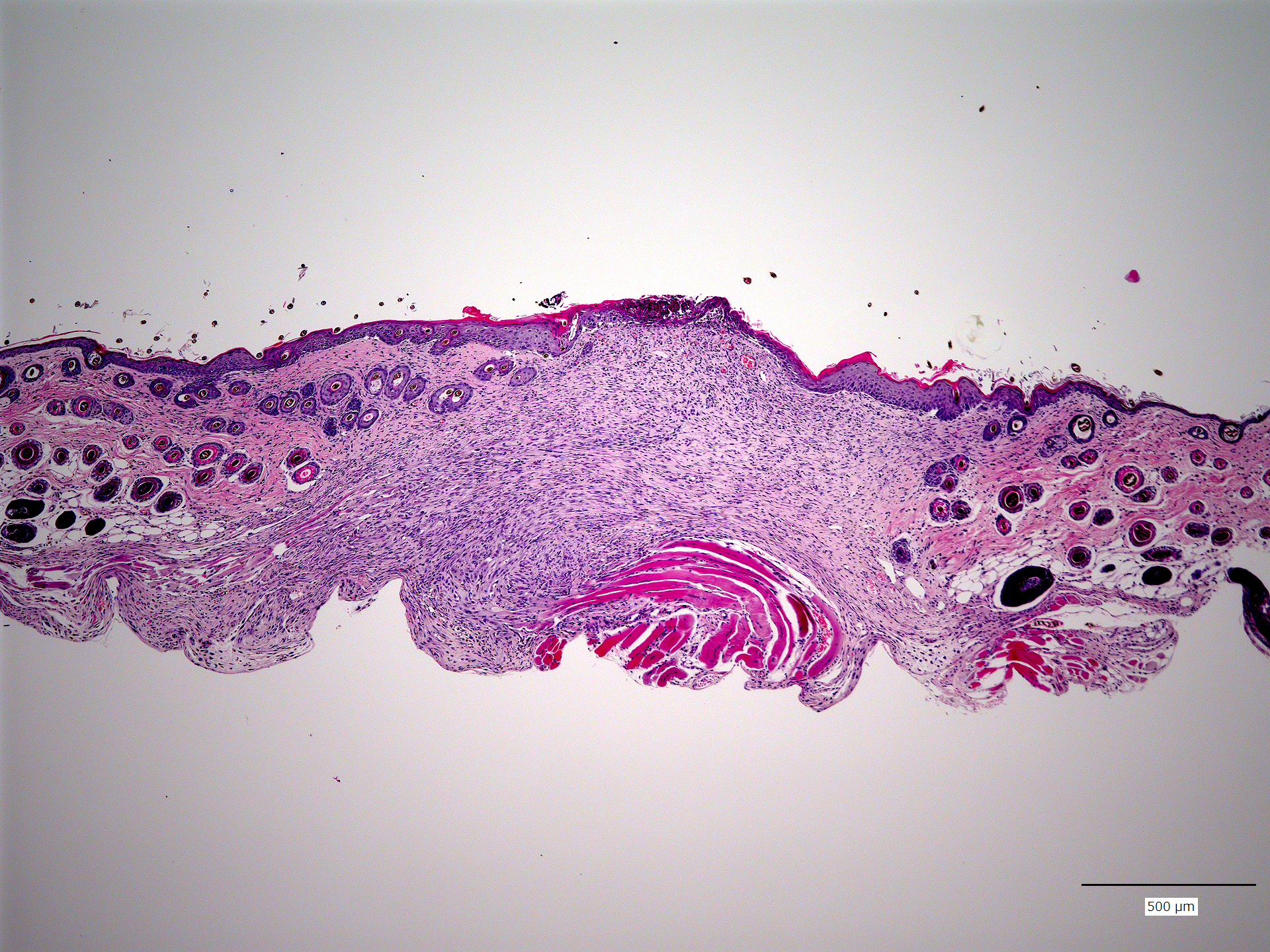

Supplement: Supplementary file 5 — Source data Fig. 1 [file 44319_2025_496_MOESM5_ESM.zip › Figure 1/1K/Day 14 Control x4.tif]

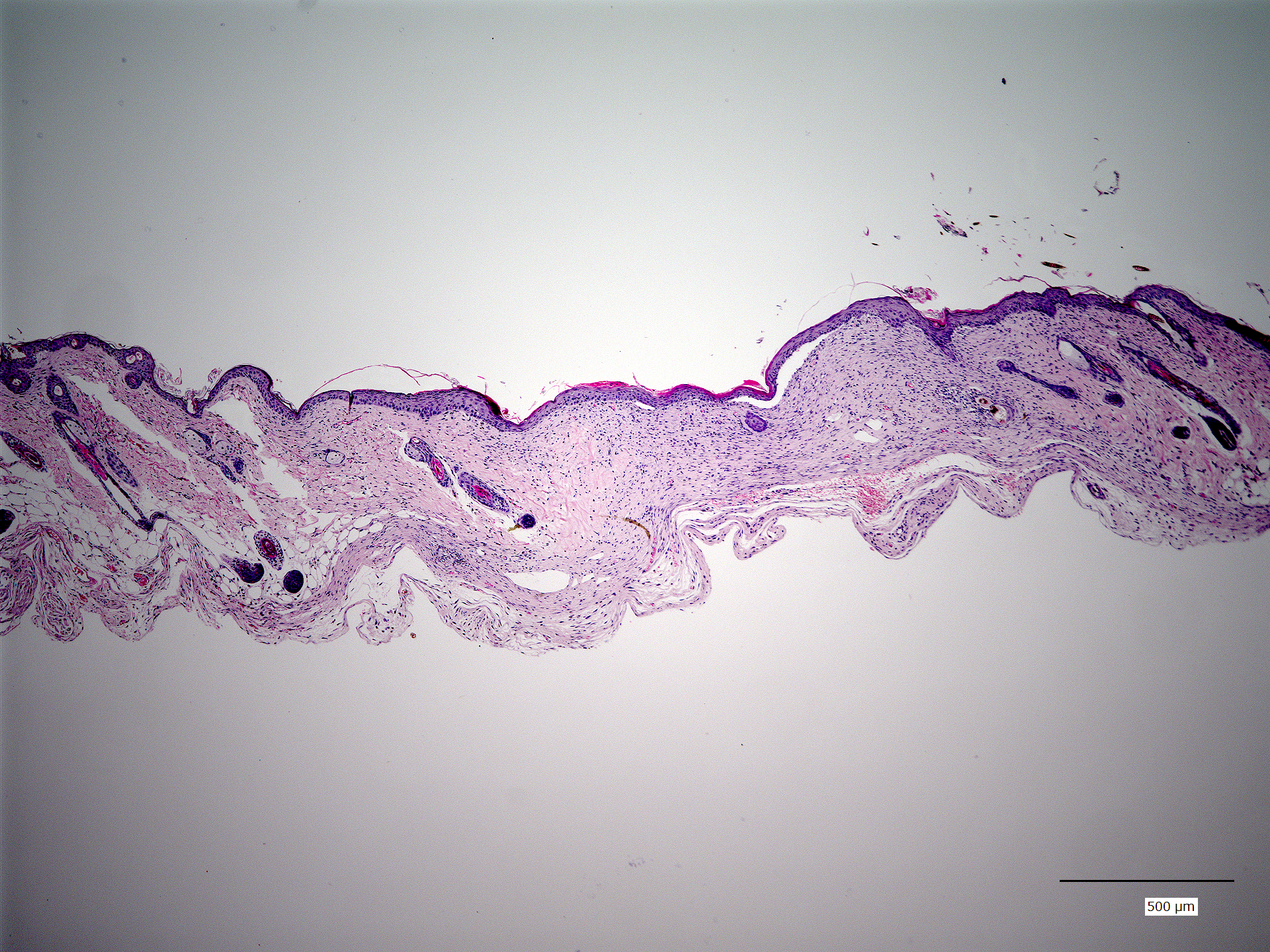

Supplement: Supplementary file 5 — Source data Fig. 1 [file 44319_2025_496_MOESM5_ESM.zip › Figure 1/1K/Day 14 KO x4 .tif]

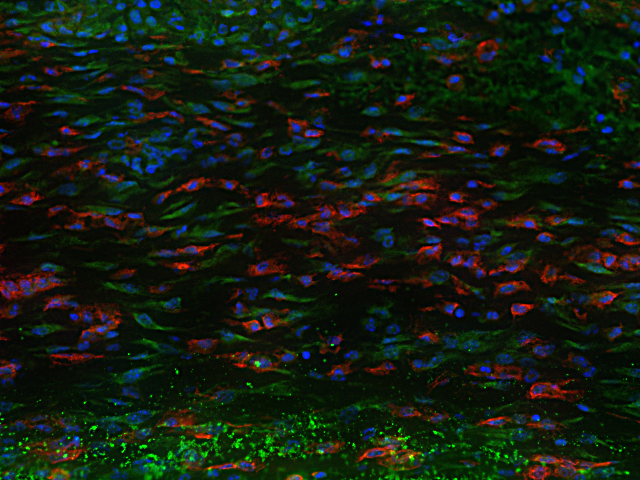

Supplement: Supplementary file 6 — Source data Fig. 5 [file 44319_2025_496_MOESM6_ESM.zip › Figure 5/5B/CTRL_x40_Fig/Figure1.tif]

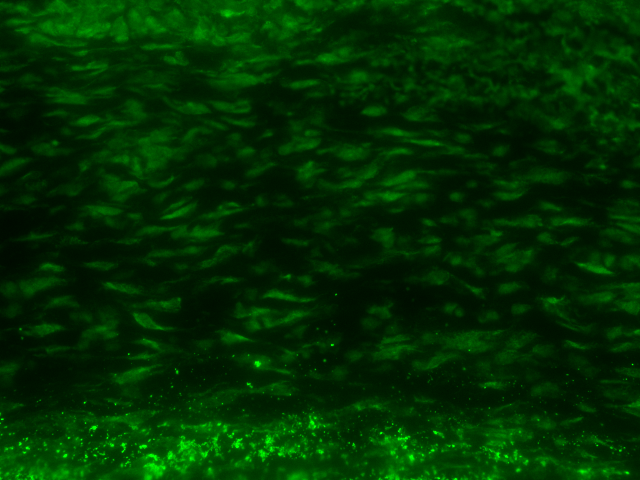

Supplement: Supplementary file 6 — Source data Fig. 5 [file 44319_2025_496_MOESM6_ESM.zip › Figure 5/5B/CTRL_x40_Fig/Image_CH1.tif]

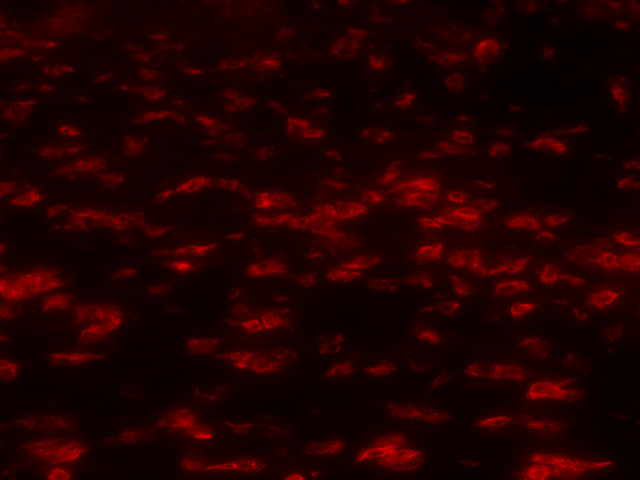

Supplement: Supplementary file 6 — Source data Fig. 5 [file 44319_2025_496_MOESM6_ESM.zip › Figure 5/5B/CTRL_x40_Fig/Image_CH2.tif]

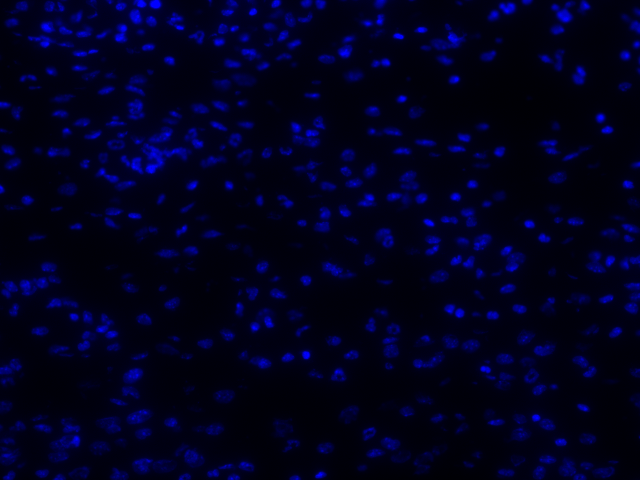

Supplement: Supplementary file 6 — Source data Fig. 5 [file 44319_2025_496_MOESM6_ESM.zip › Figure 5/5B/CTRL_x40_Fig/Image_CH3.tif]

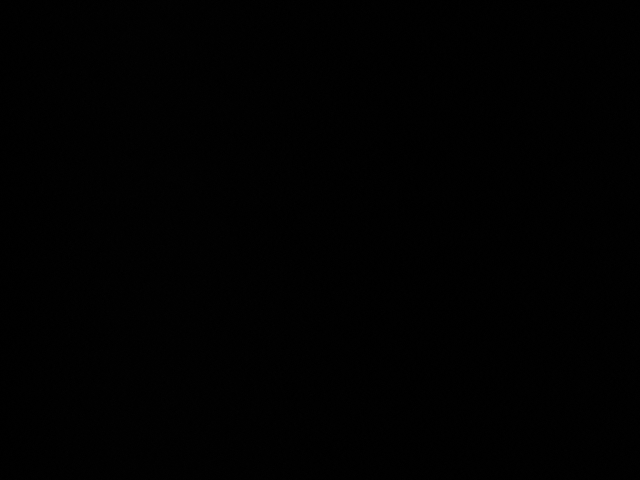

Supplement: Supplementary file 6 — Source data Fig. 5 [file 44319_2025_496_MOESM6_ESM.zip › Figure 5/5B/CTRL_x40_Fig/Image_CH4.tif]

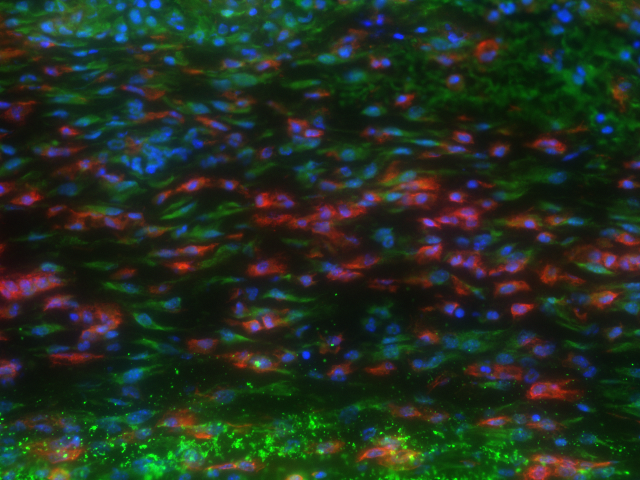

Supplement: Supplementary file 6 — Source data Fig. 5 [file 44319_2025_496_MOESM6_ESM.zip › Figure 5/5B/CTRL_x40_Fig/Image_Overlay.tif]

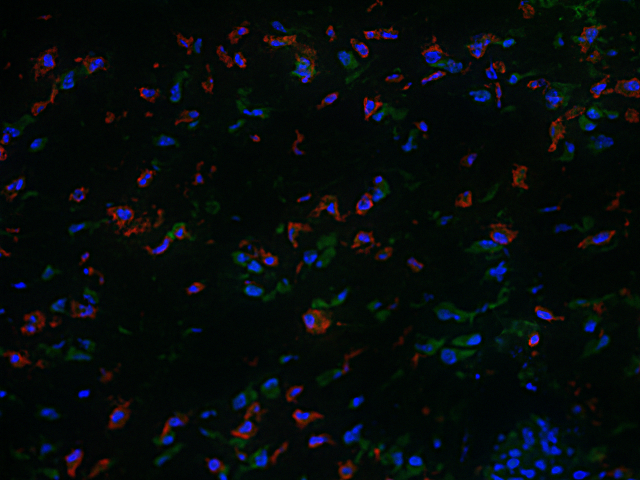

Supplement: Supplementary file 6 — Source data Fig. 5 [file 44319_2025_496_MOESM6_ESM.zip › Figure 5/5B/DTR_x40_Fig/Fig_2.tif]

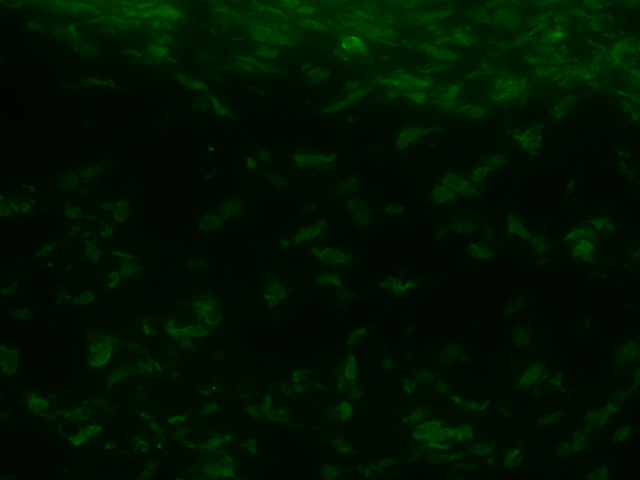

Supplement: Supplementary file 6 — Source data Fig. 5 [file 44319_2025_496_MOESM6_ESM.zip › Figure 5/5B/DTR_x40_Fig/Image_CH1.tif]

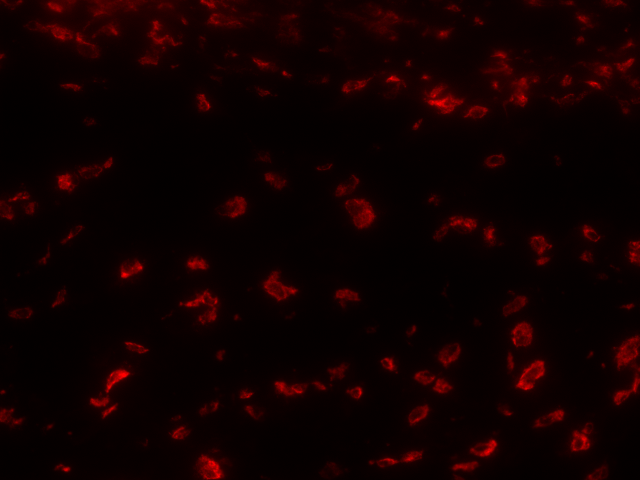

Supplement: Supplementary file 6 — Source data Fig. 5 [file 44319_2025_496_MOESM6_ESM.zip › Figure 5/5B/DTR_x40_Fig/Image_CH2.tif]

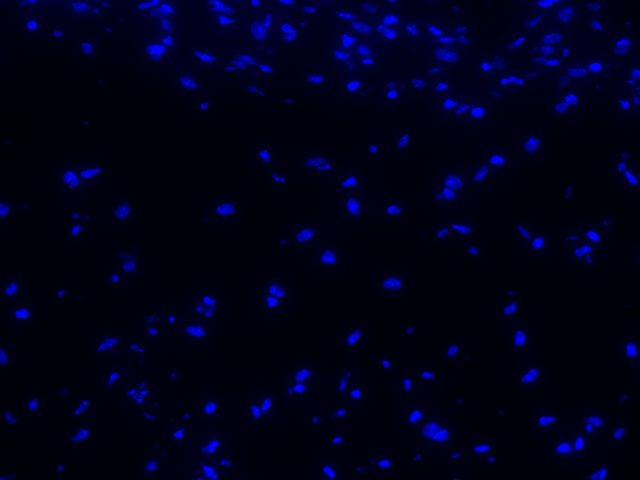

Supplement: Supplementary file 6 — Source data Fig. 5 [file 44319_2025_496_MOESM6_ESM.zip › Figure 5/5B/DTR_x40_Fig/Image_CH3.tif]

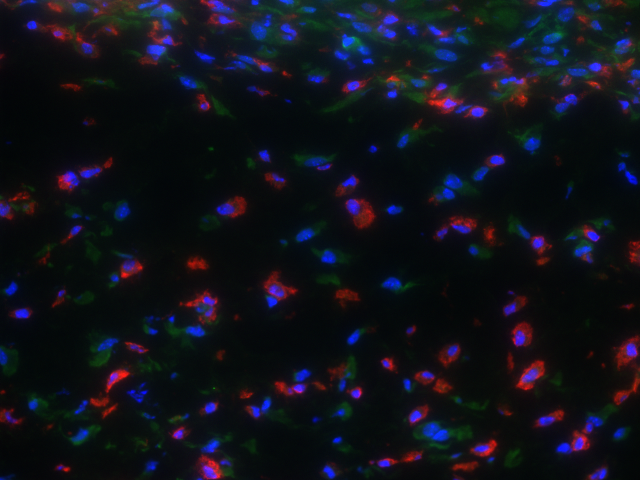

Supplement: Supplementary file 6 — Source data Fig. 5 [file 44319_2025_496_MOESM6_ESM.zip › Figure 5/5B/DTR_x40_Fig/Image_Overlay.tif]

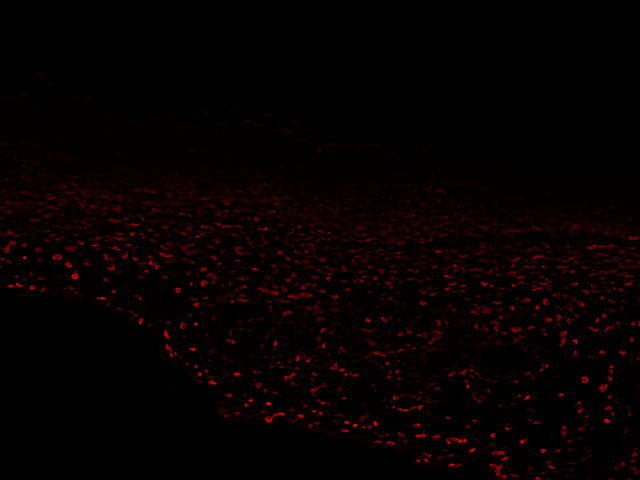

Supplement: Supplementary file 6 — Source data Fig. 5 [file 44319_2025_496_MOESM6_ESM.zip › Figure 5/5B/low_magnification_Fig/CTRL_CD206.tif]

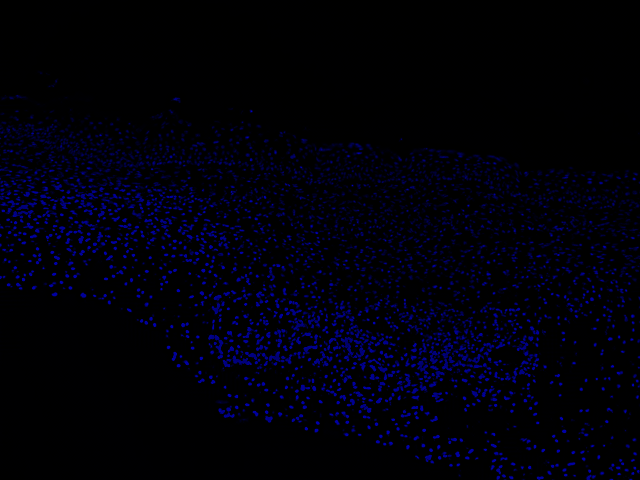

Supplement: Supplementary file 6 — Source data Fig. 5 [file 44319_2025_496_MOESM6_ESM.zip › Figure 5/5B/low_magnification_Fig/CTRL_DAPI.tif]

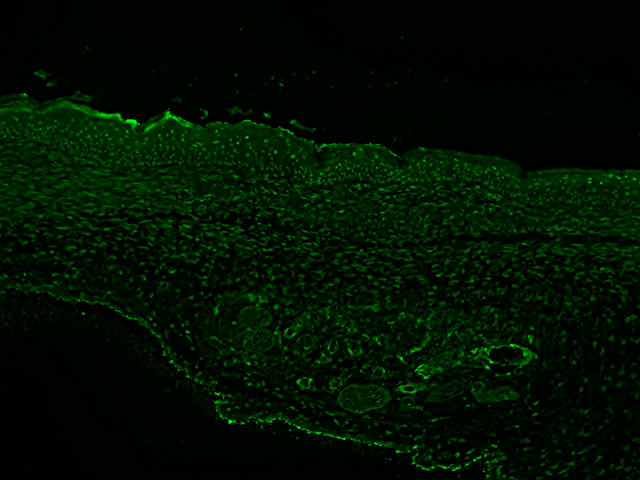

Supplement: Supplementary file 6 — Source data Fig. 5 [file 44319_2025_496_MOESM6_ESM.zip › Figure 5/5B/low_magnification_Fig/CTRL_En1.tif]

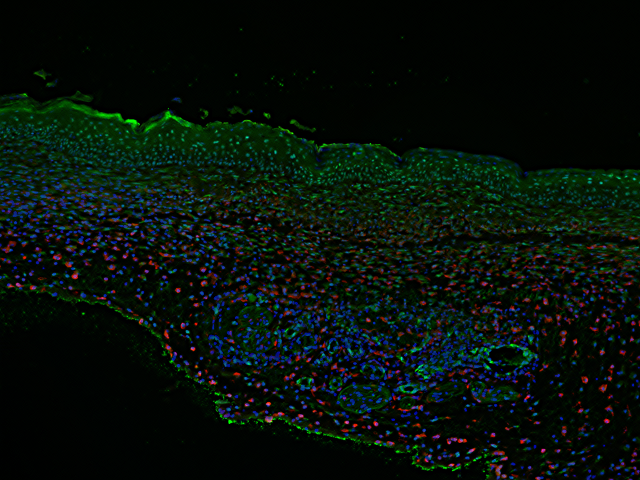

Supplement: Supplementary file 6 — Source data Fig. 5 [file 44319_2025_496_MOESM6_ESM.zip › Figure 5/5B/low_magnification_Fig/CTRL_overlay.tif]

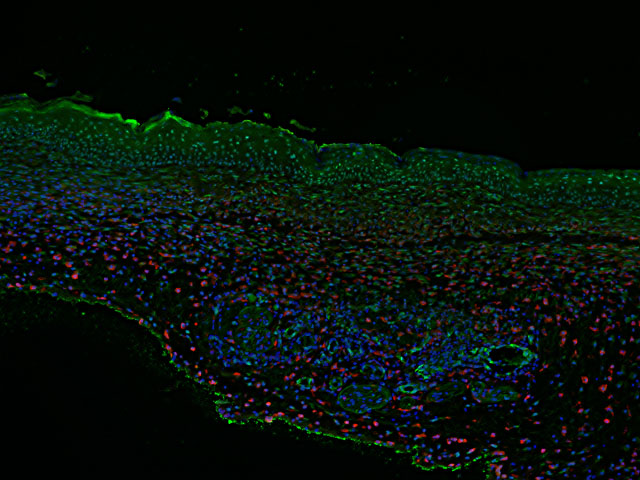

Supplement: Supplementary file 6 — Source data Fig. 5 [file 44319_2025_496_MOESM6_ESM.zip › Figure 5/5B/low_magnification_Fig/CTRL_overlay_JPEG.jpg]

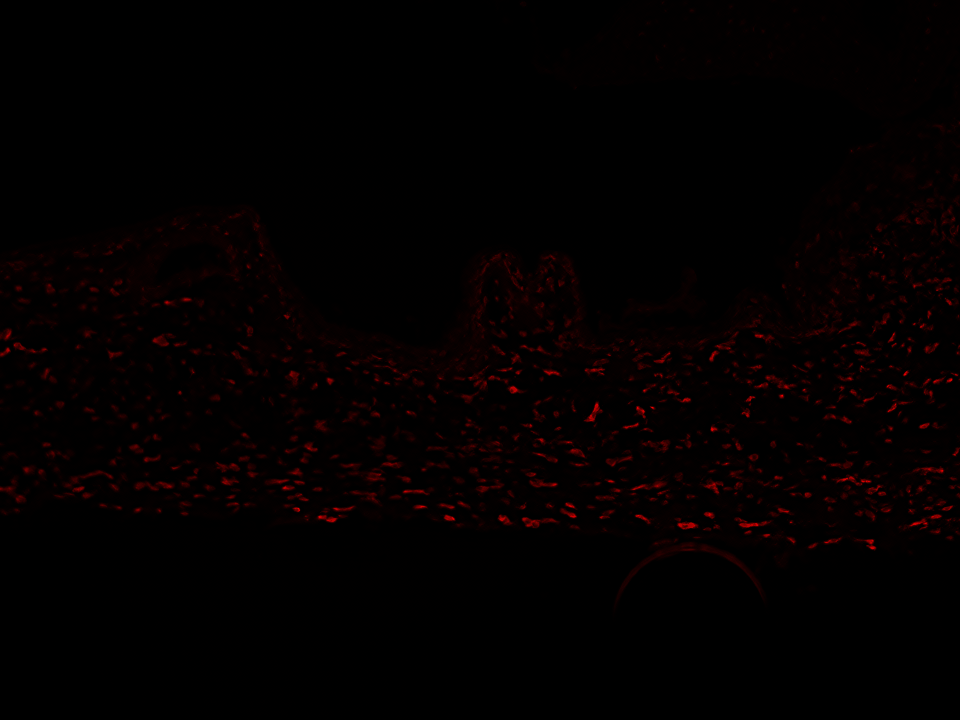

Supplement: Supplementary file 6 — Source data Fig. 5 [file 44319_2025_496_MOESM6_ESM.zip › Figure 5/5B/low_magnification_Fig/DTR_CD206.tif]

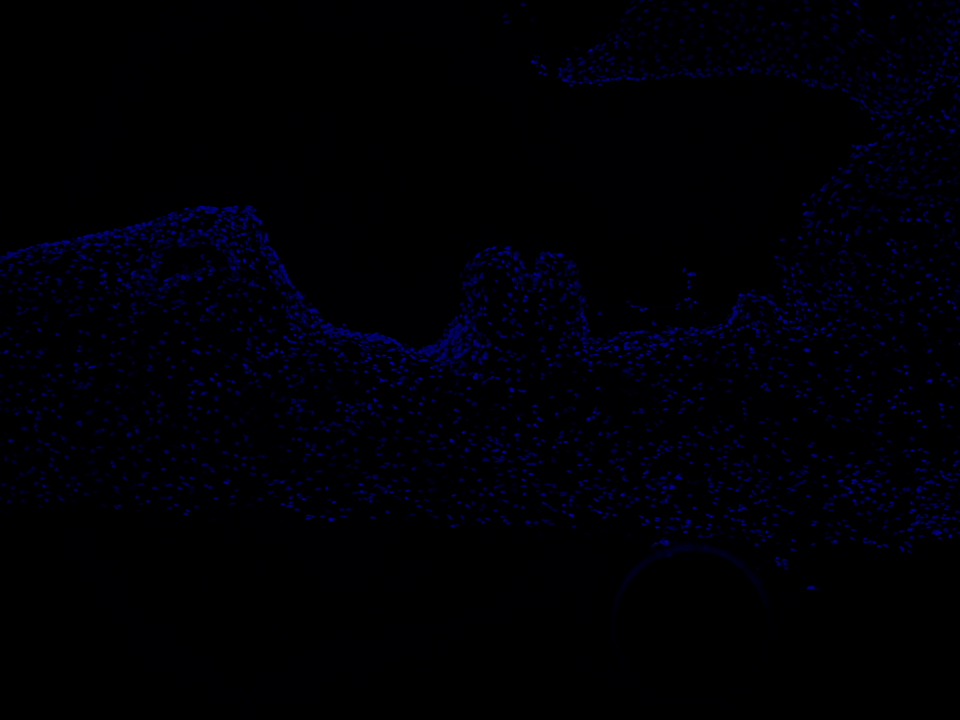

Supplement: Supplementary file 6 — Source data Fig. 5 [file 44319_2025_496_MOESM6_ESM.zip › Figure 5/5B/low_magnification_Fig/DTR_DAPI.tif]

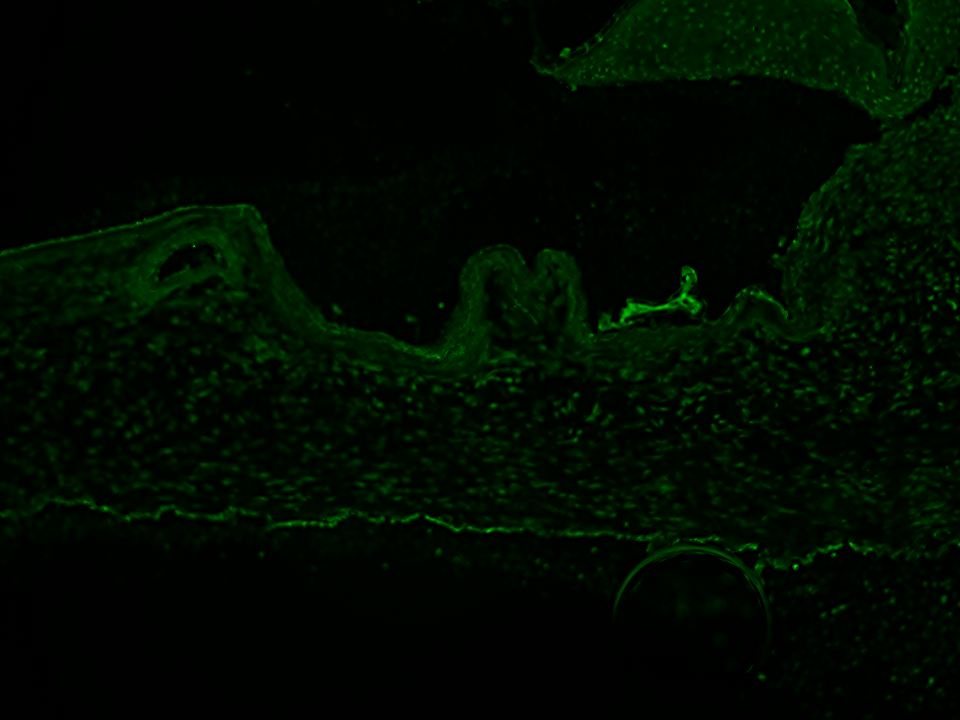

Supplement: Supplementary file 6 — Source data Fig. 5 [file 44319_2025_496_MOESM6_ESM.zip › Figure 5/5B/low_magnification_Fig/DTR_En1.tif]

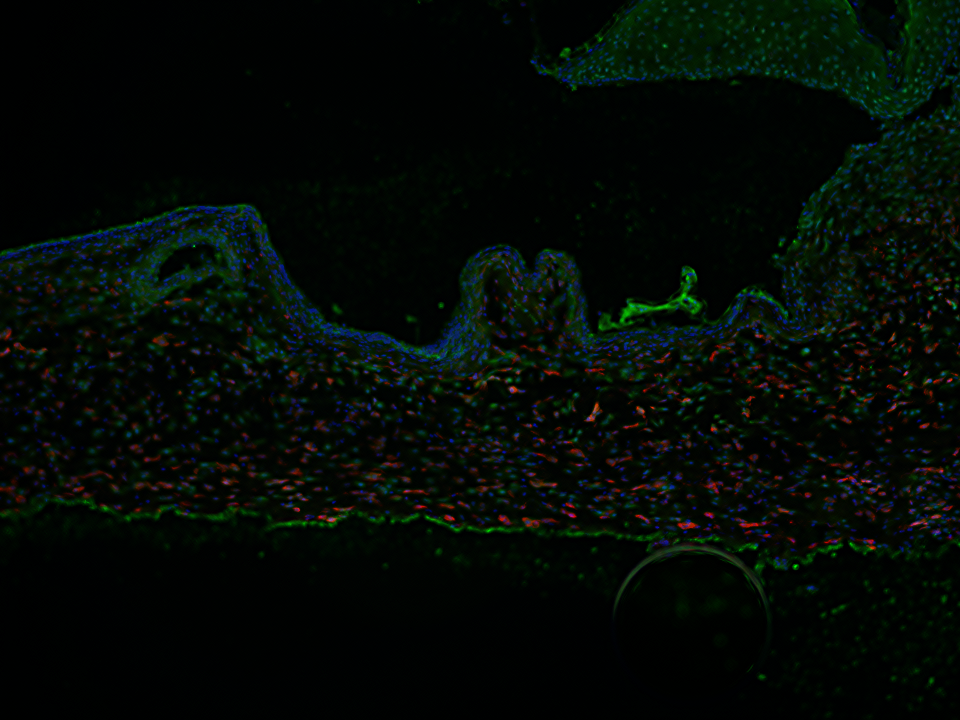

Supplement: Supplementary file 6 — Source data Fig. 5 [file 44319_2025_496_MOESM6_ESM.zip › Figure 5/5B/low_magnification_Fig/DTR_overlay.tif]

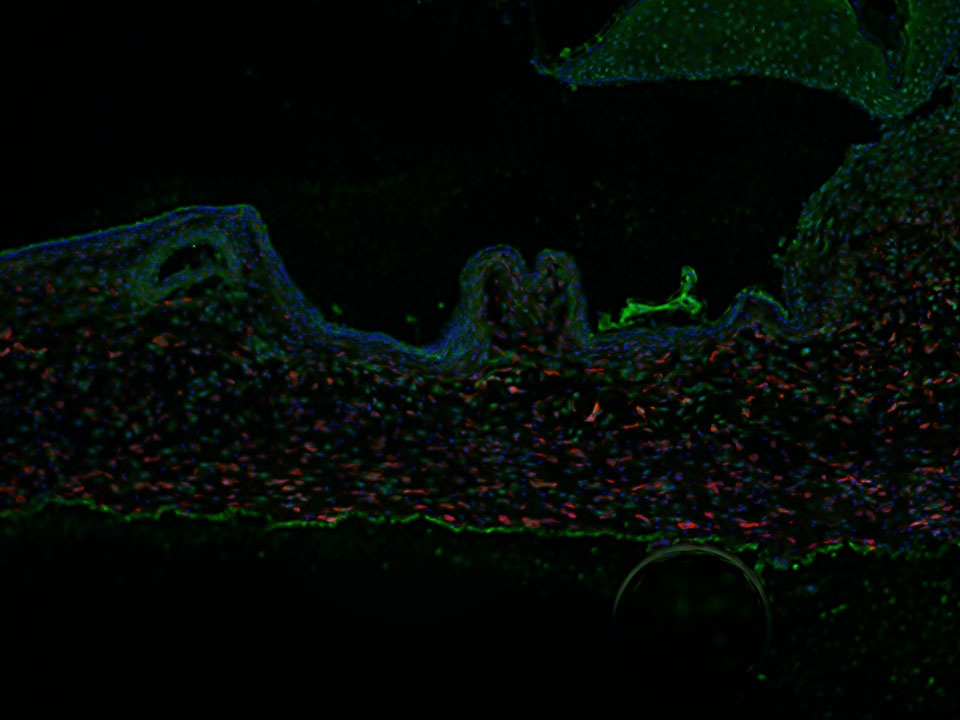

Supplement: Supplementary file 6 — Source data Fig. 5 [file 44319_2025_496_MOESM6_ESM.zip › Figure 5/5B/low_magnification_Fig/DTR_overlay_JPEG.jpg]

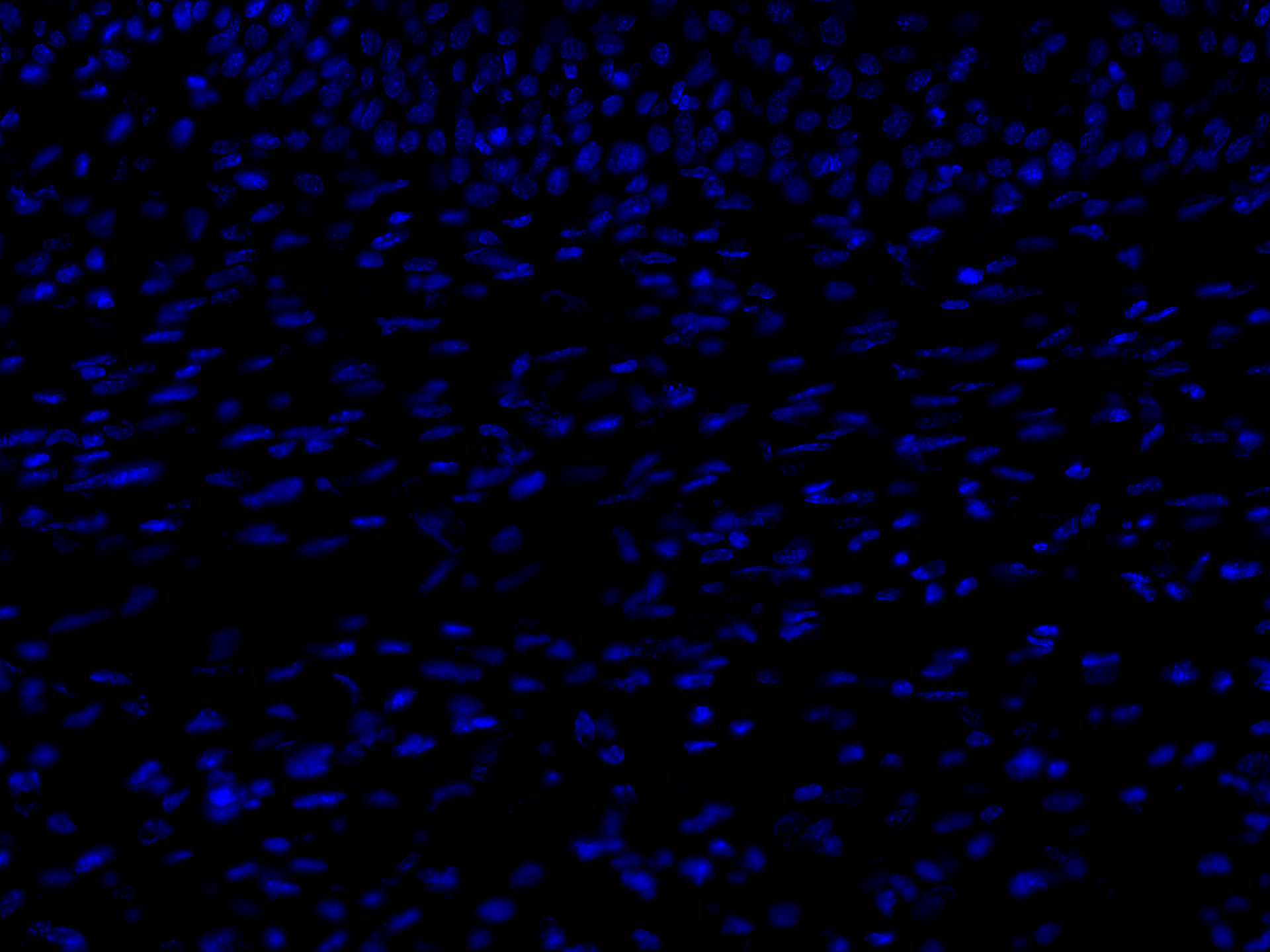

Supplement: Supplementary file 6 — Source data Fig. 5 [file 44319_2025_496_MOESM6_ESM.zip › Figure 5/5D/Ctrl/DAPI.tif]

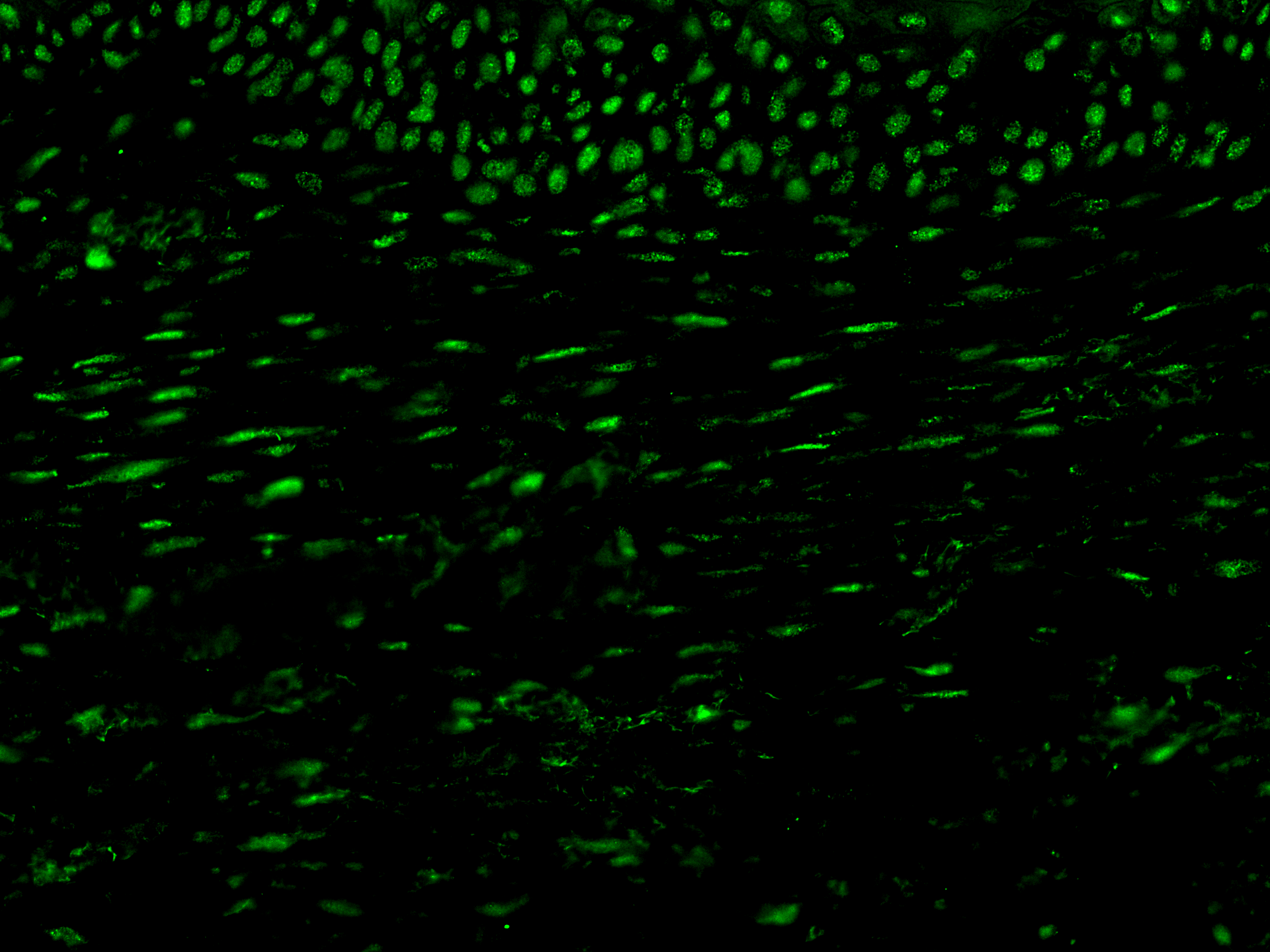

Supplement: Supplementary file 6 — Source data Fig. 5 [file 44319_2025_496_MOESM6_ESM.zip › Figure 5/5D/Ctrl/EN1.tif]

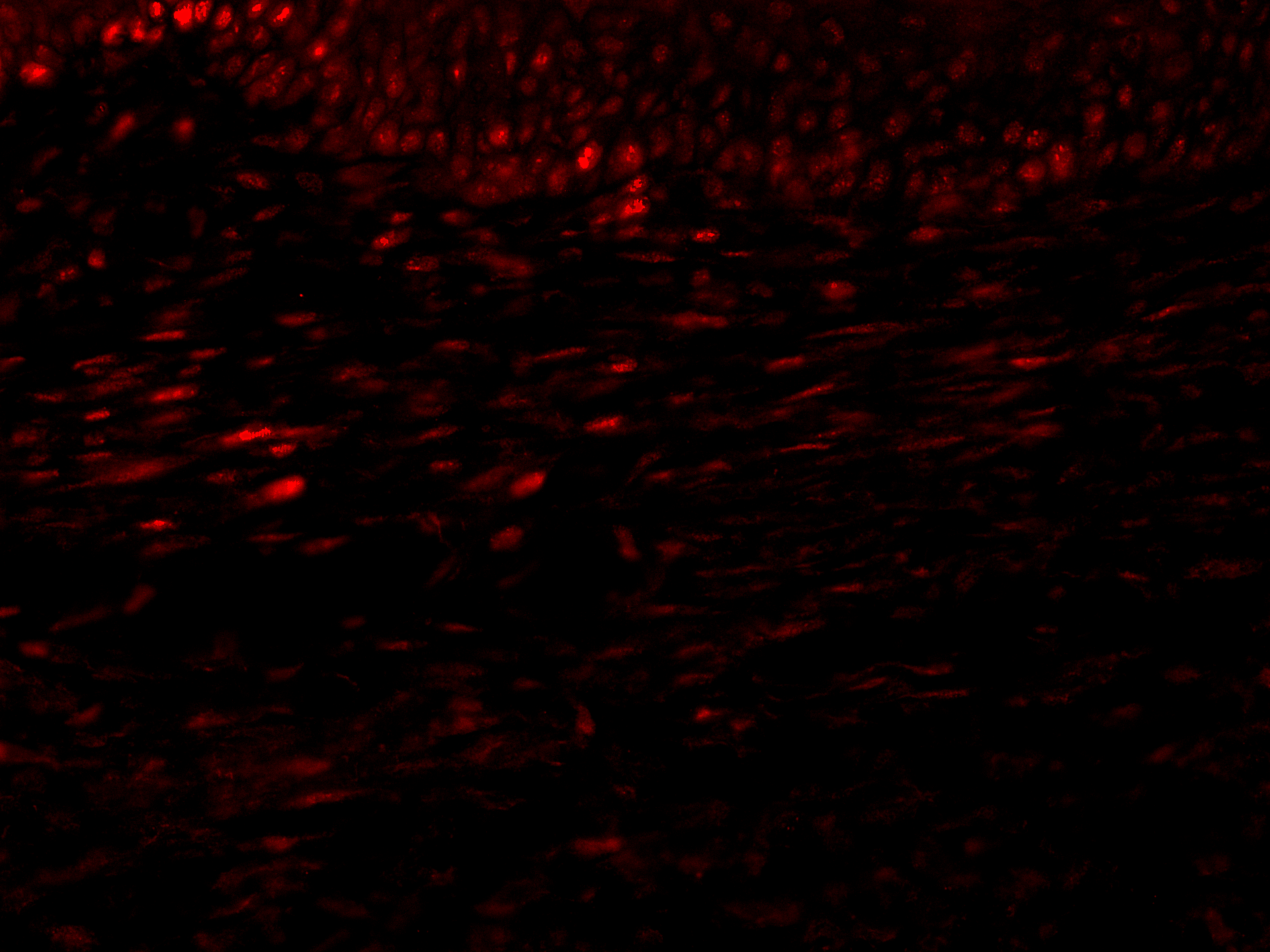

Supplement: Supplementary file 6 — Source data Fig. 5 [file 44319_2025_496_MOESM6_ESM.zip › Figure 5/5D/Ctrl/Ki67.tif]

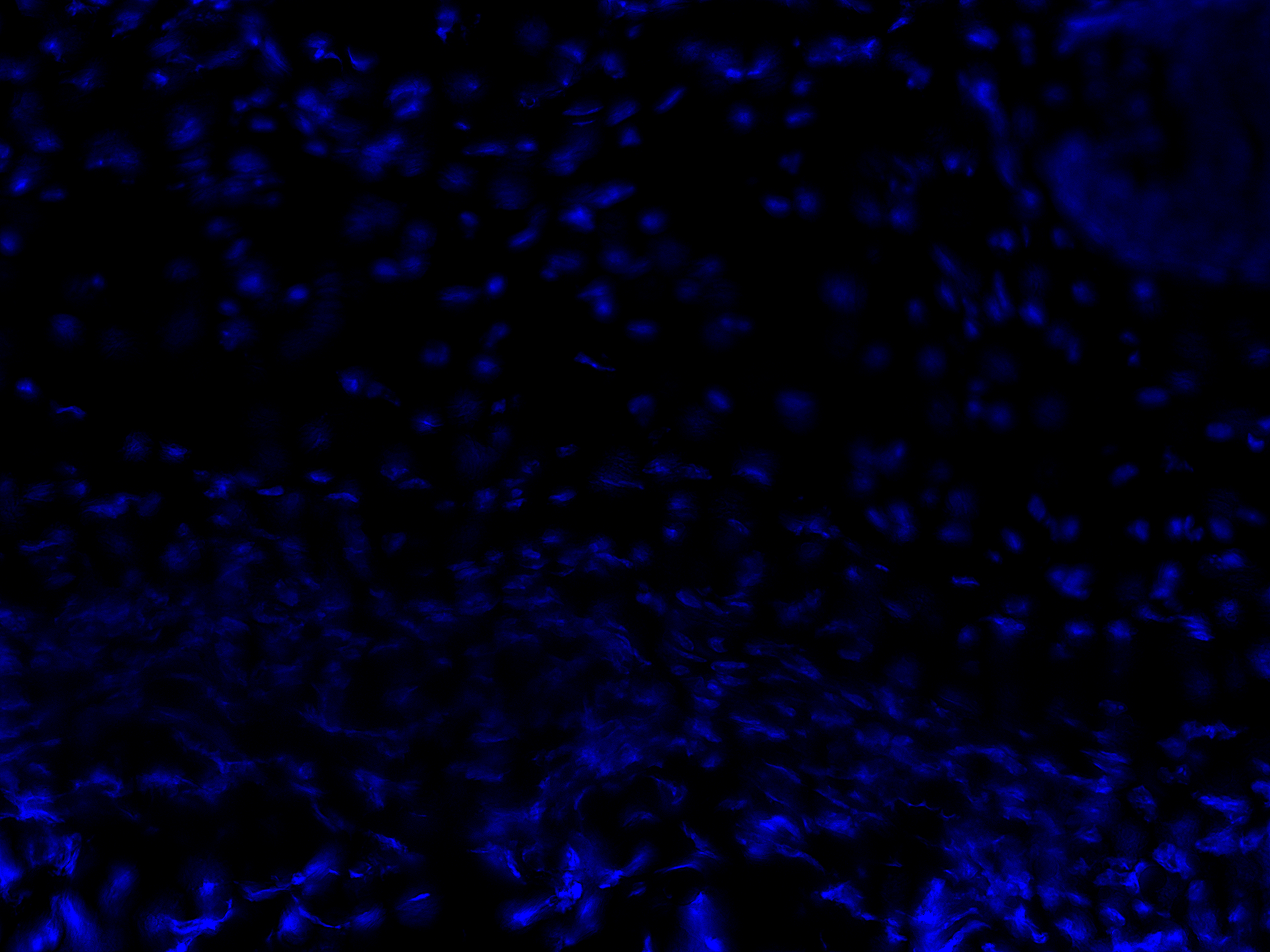

Supplement: Supplementary file 6 — Source data Fig. 5 [file 44319_2025_496_MOESM6_ESM.zip › Figure 5/5D/Mrc1-DTR/DAPI.tif]

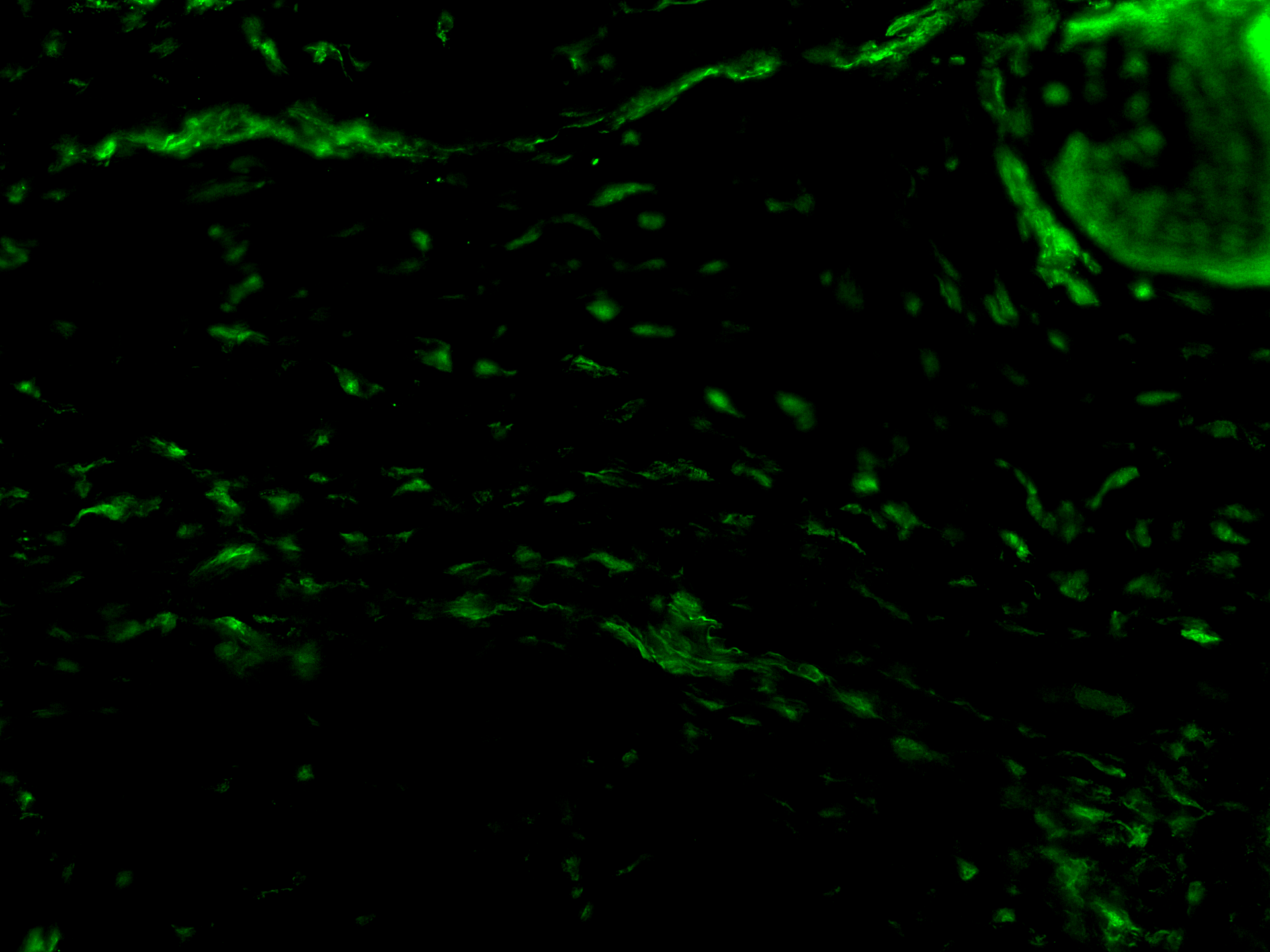

Supplement: Supplementary file 6 — Source data Fig. 5 [file 44319_2025_496_MOESM6_ESM.zip › Figure 5/5D/Mrc1-DTR/EN1.tif]

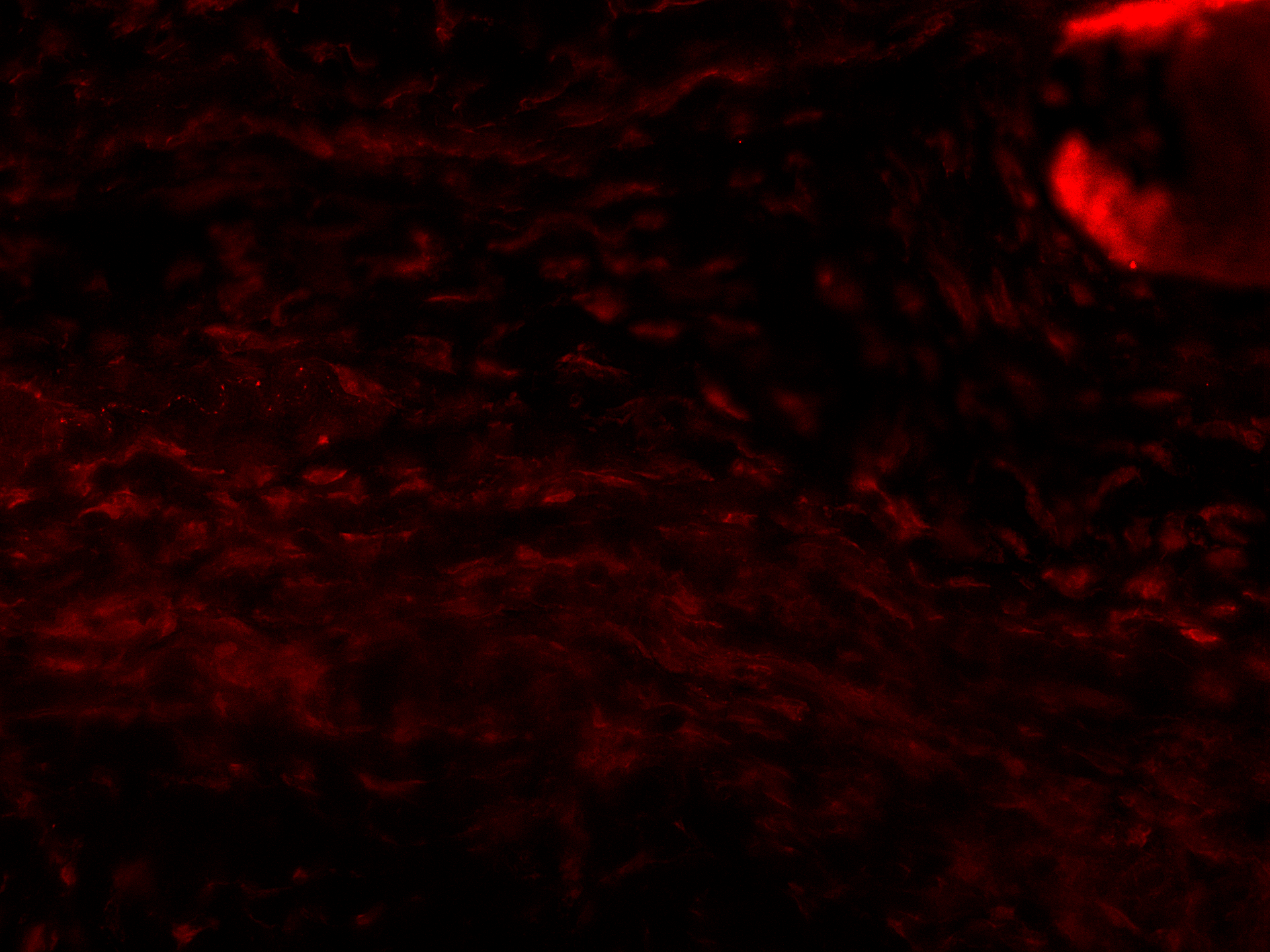

Supplement: Supplementary file 6 — Source data Fig. 5 [file 44319_2025_496_MOESM6_ESM.zip › Figure 5/5D/Mrc1-DTR/Ki67.tif]

## Slide 1
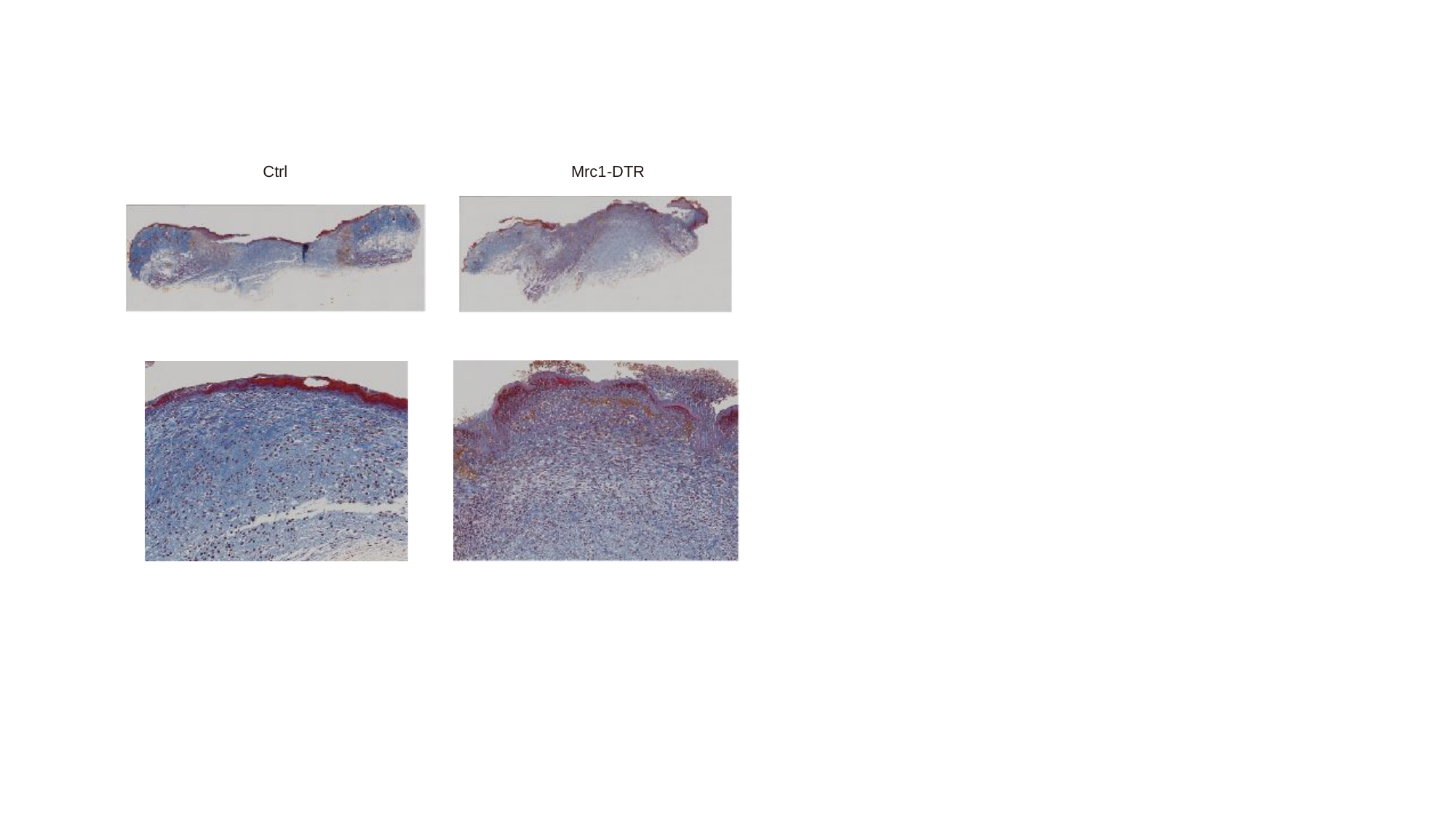

Ctrl
Mrc1
-
DTR

Supplement: Supplementary file 6 — Source data Fig. 5 [file 44319_2025_496_MOESM6_ESM.zip › Figure 5/Fig. 5F.pptx]

## Slide 1
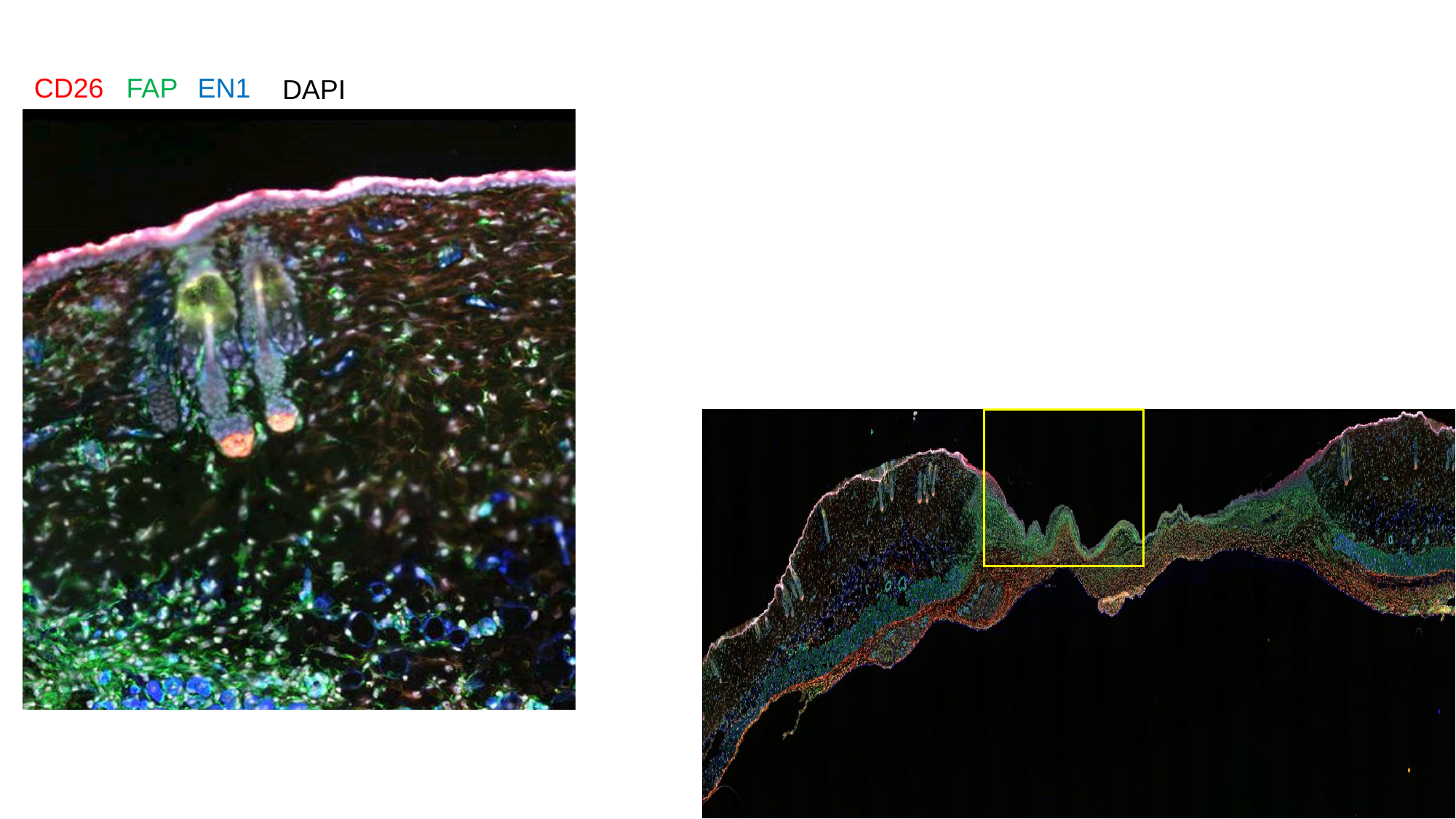

CD26
FAP
EN1
DAPI

## Slide 2
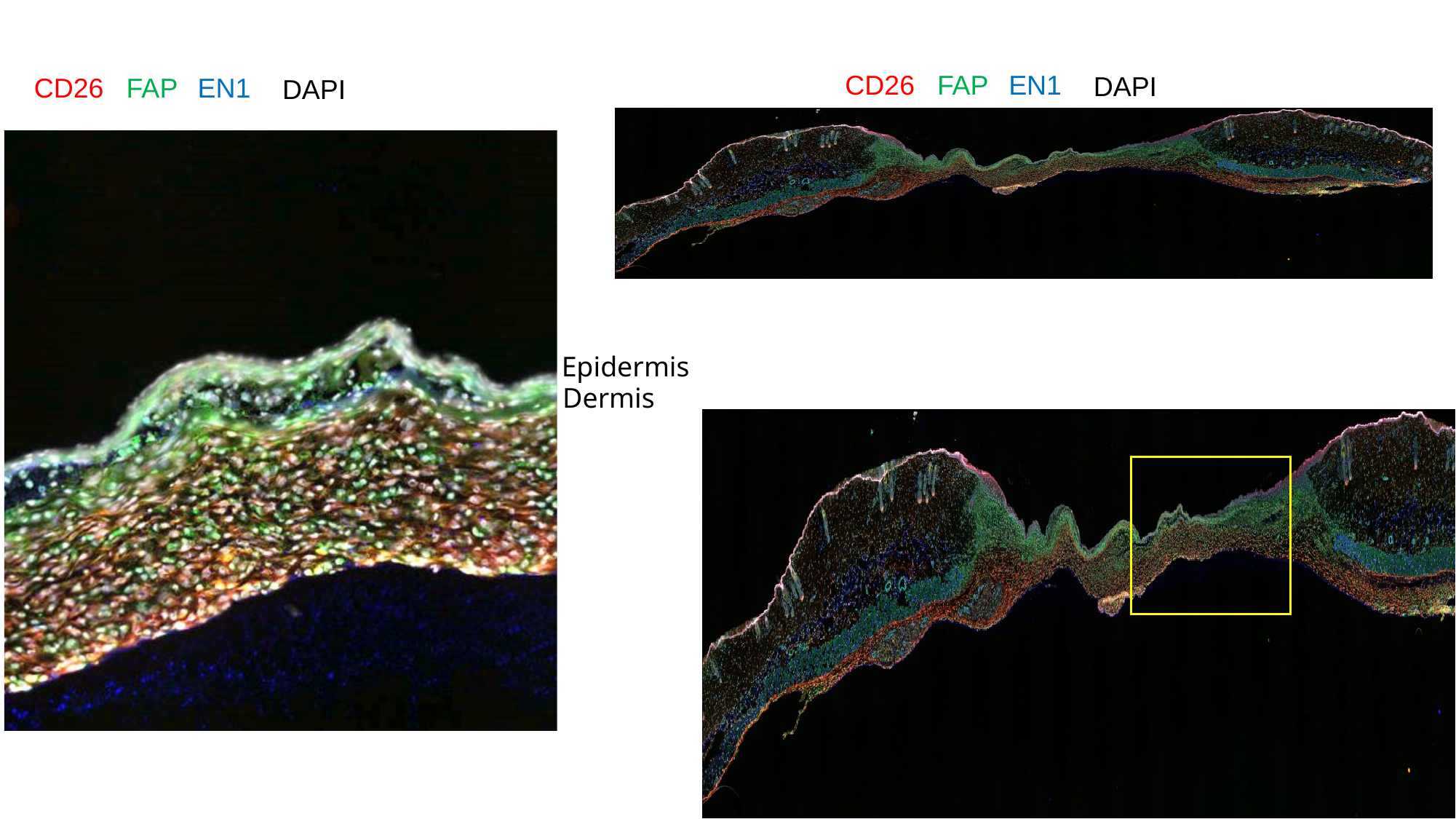

CD26
FAP
EN1
DAPI
CD26
FAP
EN1
DAPI
Epidermis
Dermis

Supplement: Supplementary file 6 — Source data Fig. 5 [file 44319_2025_496_MOESM6_ESM.zip › Figure 5/Fig.5A.pptx]

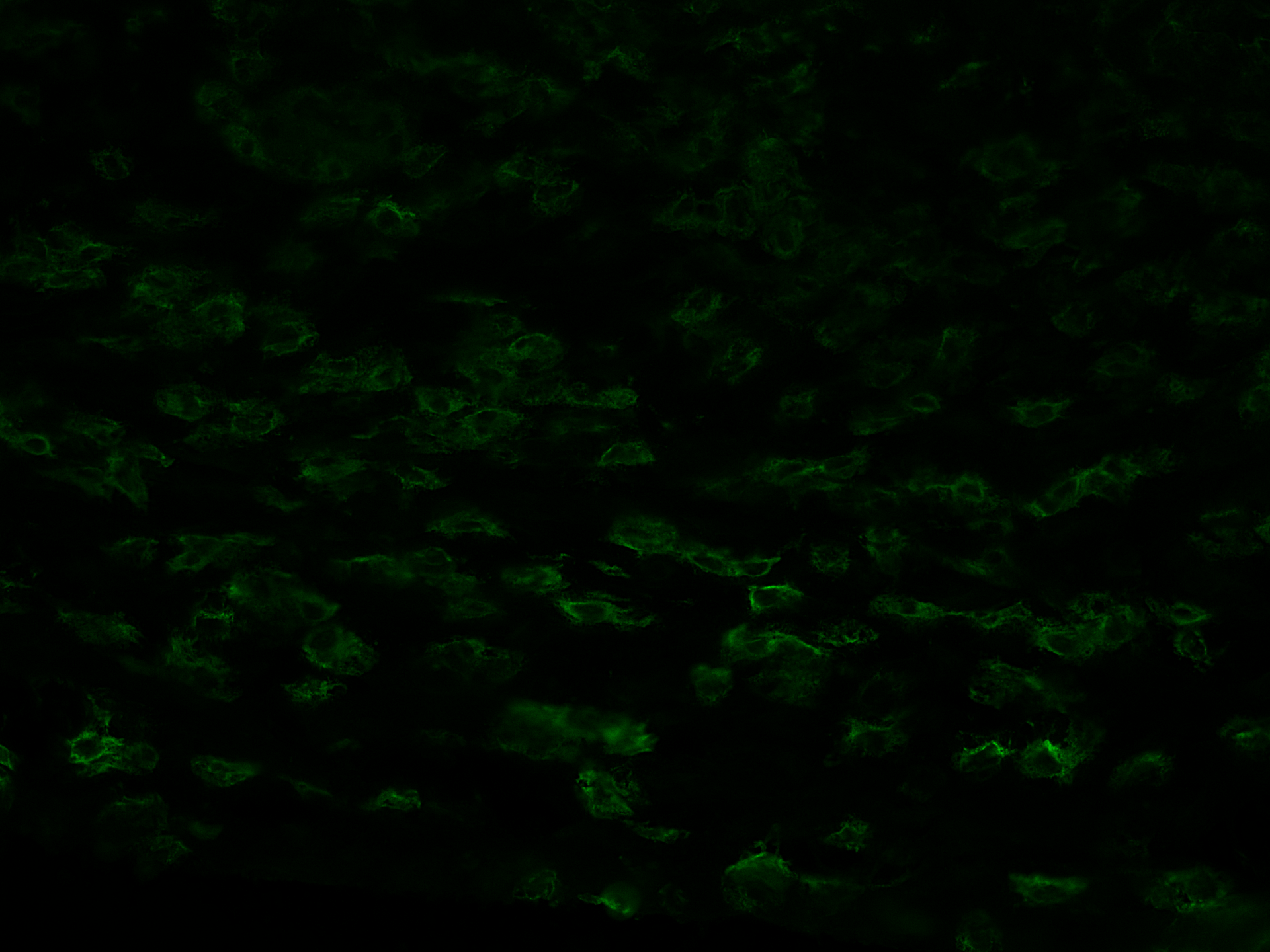

Supplement: Supplementary file 7 — Source data Fig. 6 [file 44319_2025_496_MOESM7_ESM.zip › Figure 6/6B/20230821PDGFA_Fig/CTRL_CD206.tif]

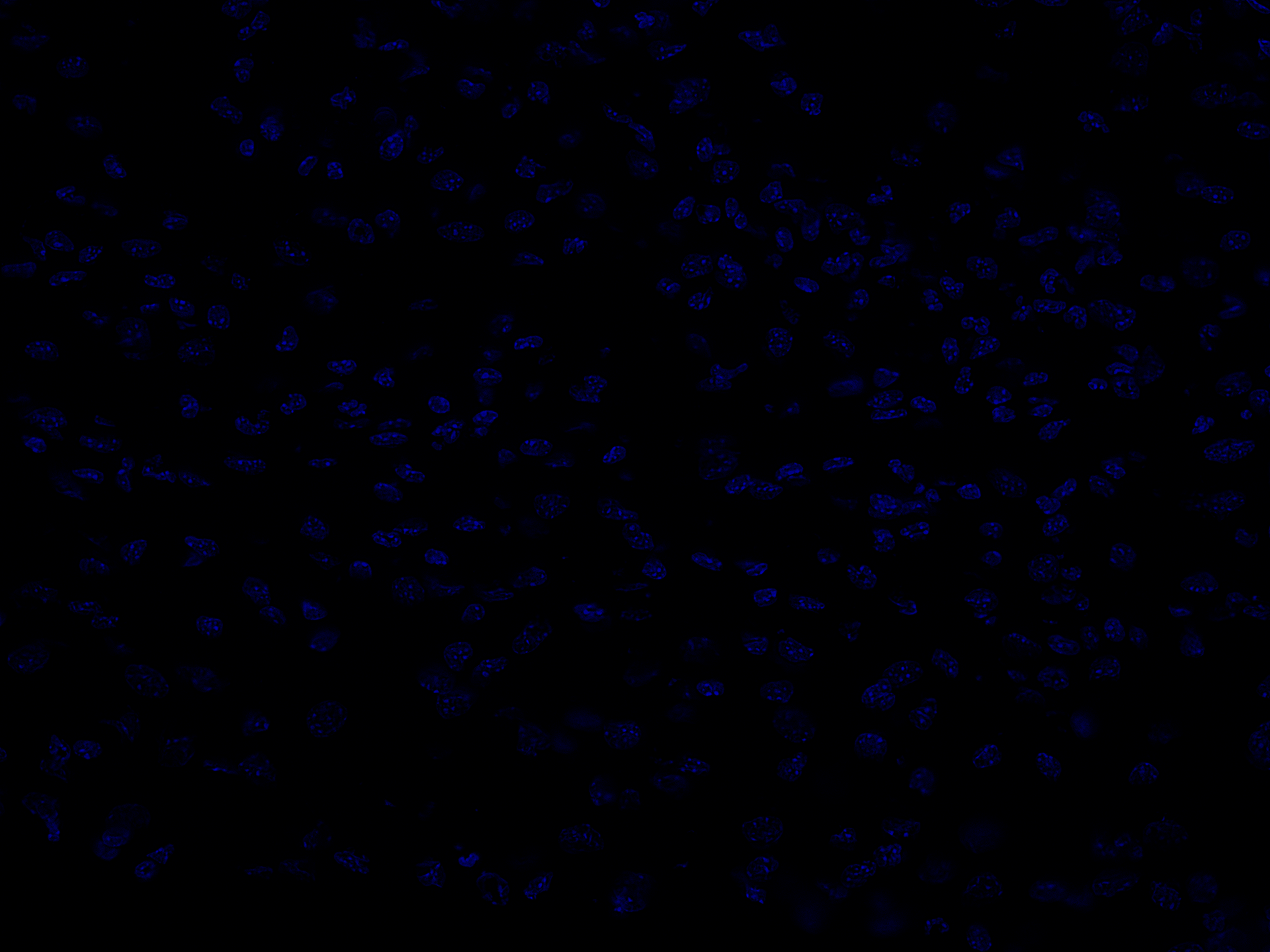

Supplement: Supplementary file 7 — Source data Fig. 6 [file 44319_2025_496_MOESM7_ESM.zip › Figure 6/6B/20230821PDGFA_Fig/CTRL_DAPI.tif]

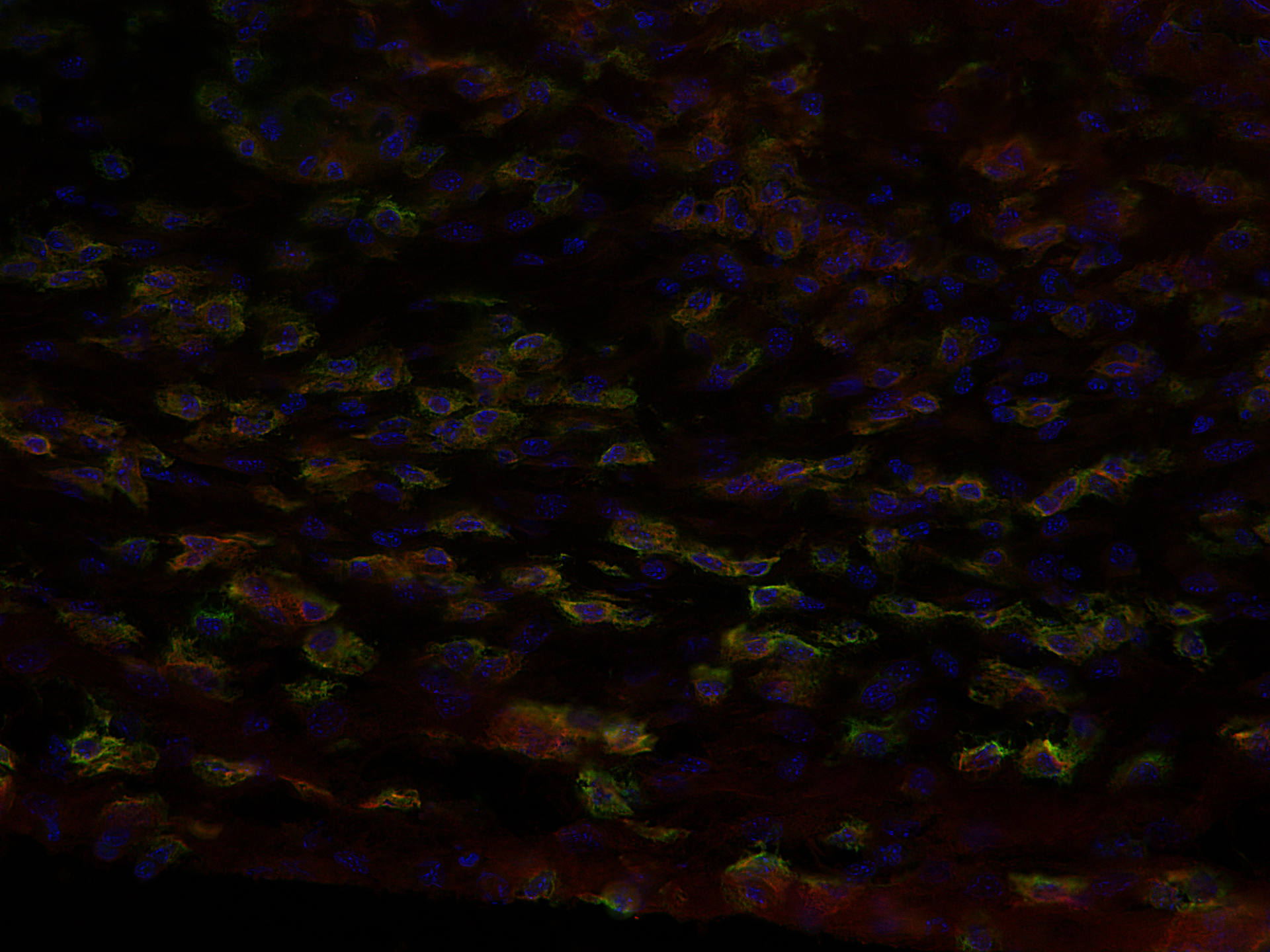

Supplement: Supplementary file 7 — Source data Fig. 6 [file 44319_2025_496_MOESM7_ESM.zip › Figure 6/6B/20230821PDGFA_Fig/CTRL_overlay.tif]

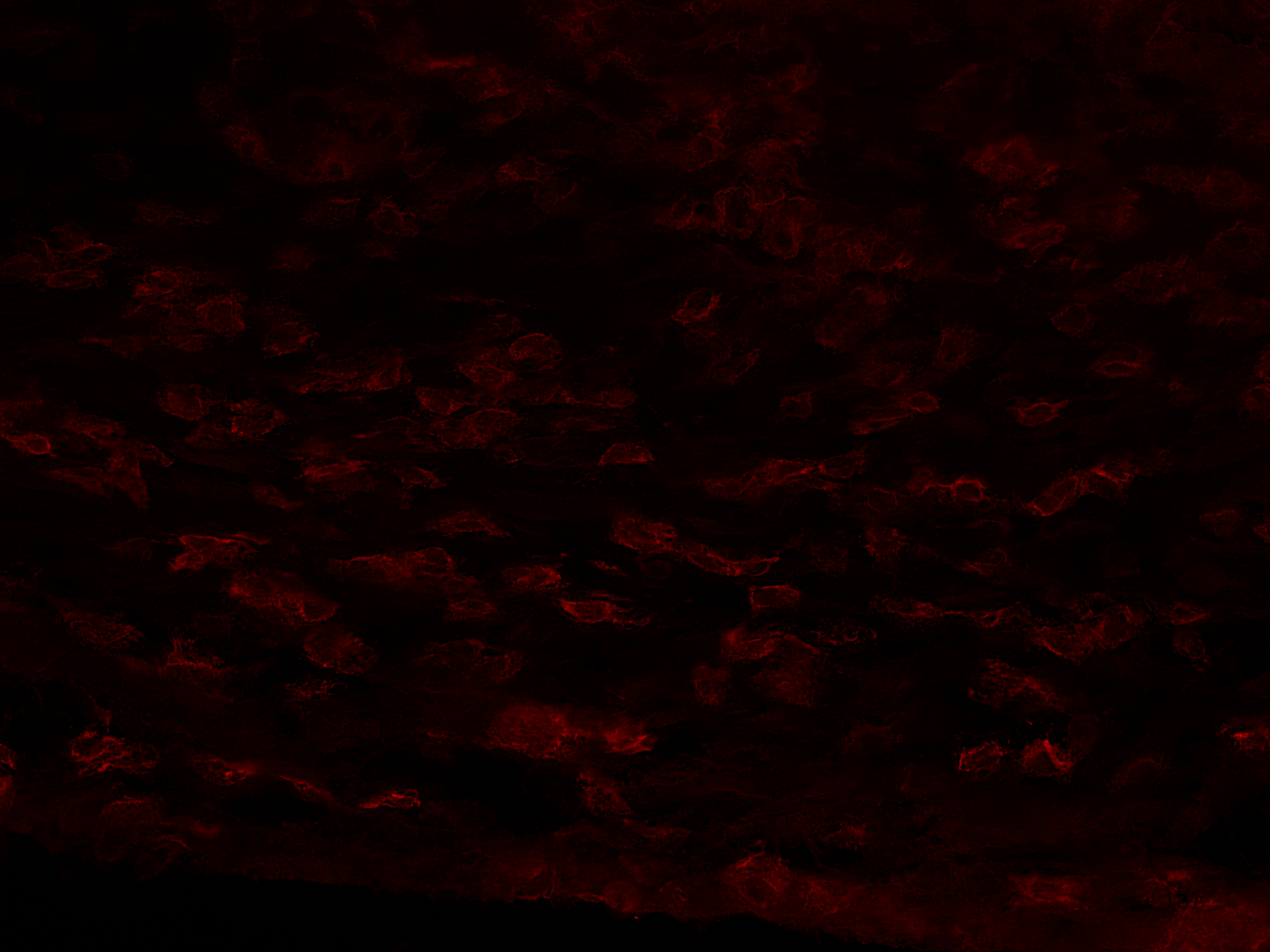

Supplement: Supplementary file 7 — Source data Fig. 6 [file 44319_2025_496_MOESM7_ESM.zip › Figure 6/6B/20230821PDGFA_Fig/CTRL_PDGFA.tif]

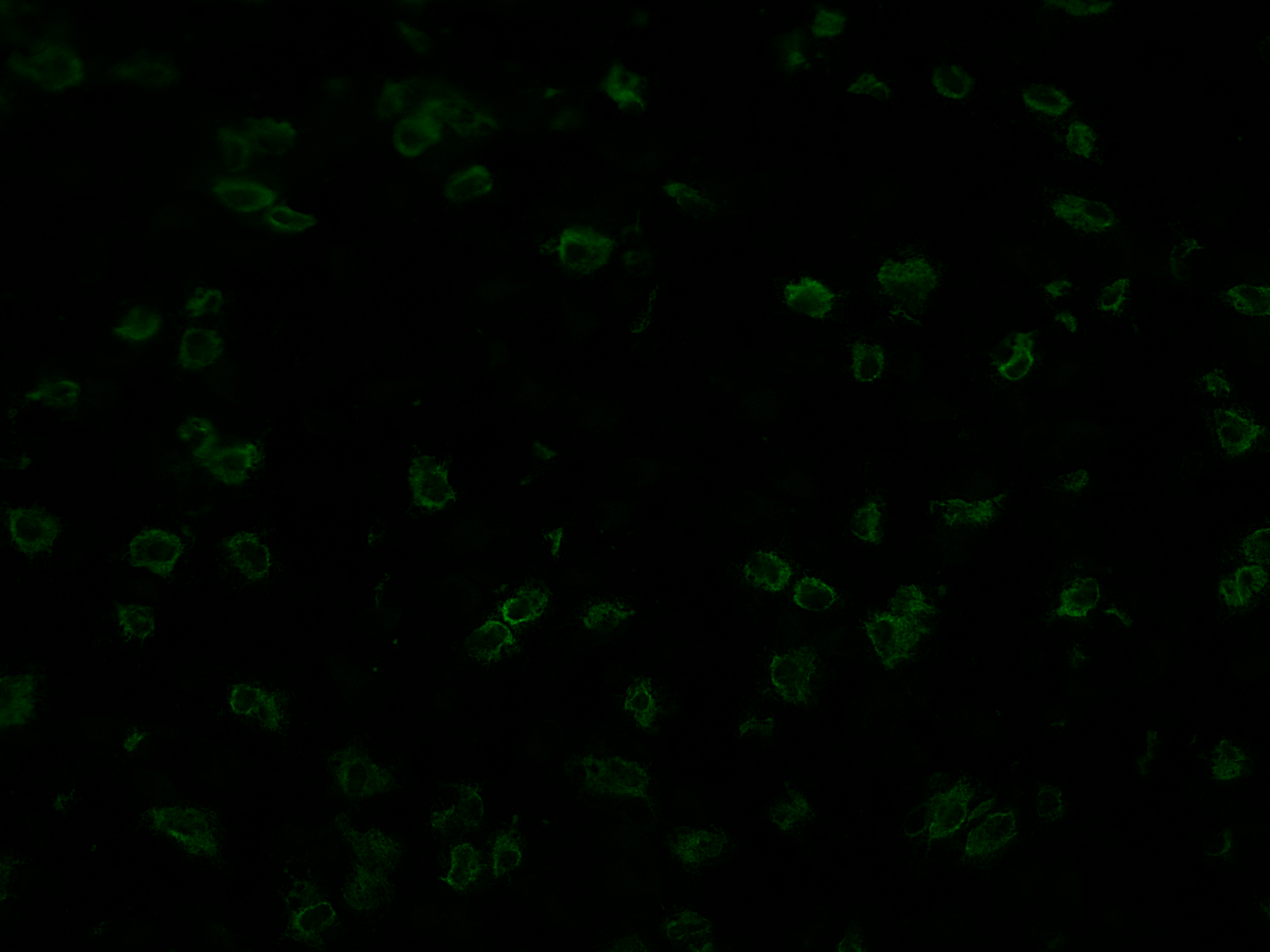

Supplement: Supplementary file 7 — Source data Fig. 6 [file 44319_2025_496_MOESM7_ESM.zip › Figure 6/6B/20230821PDGFA_Fig/DTR_CD206.tif]

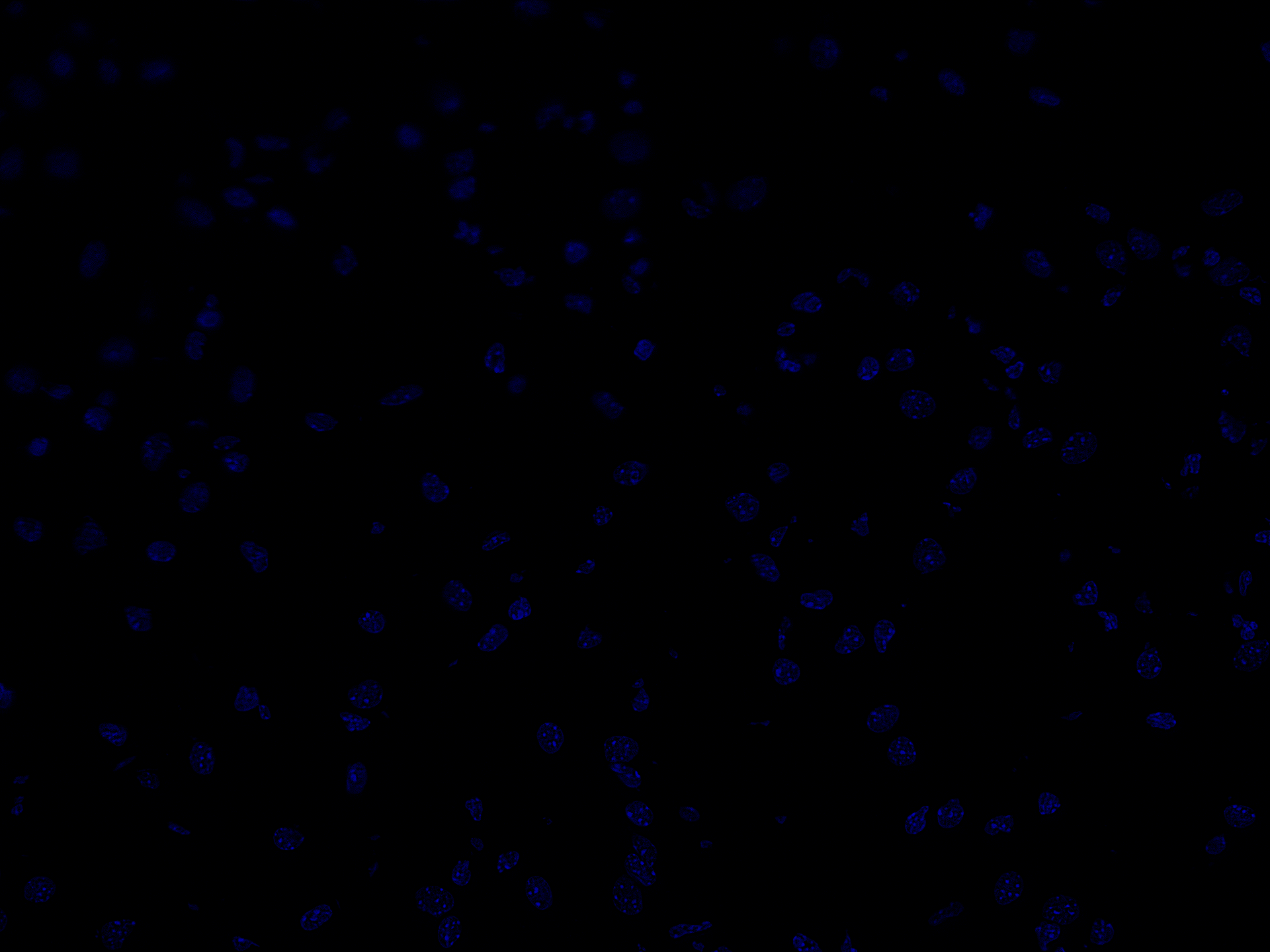

Supplement: Supplementary file 7 — Source data Fig. 6 [file 44319_2025_496_MOESM7_ESM.zip › Figure 6/6B/20230821PDGFA_Fig/DTR_DAPI.tif]

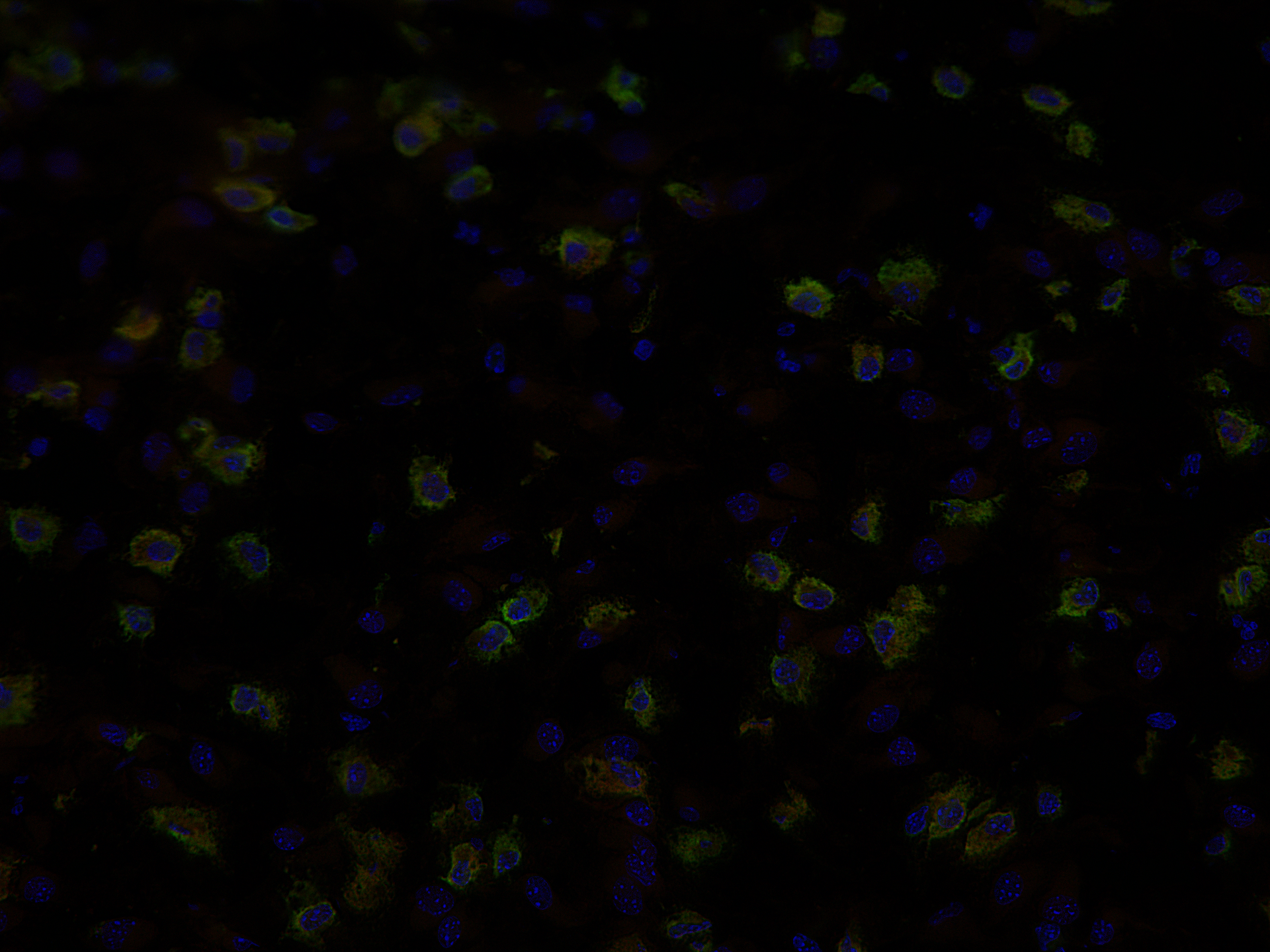

Supplement: Supplementary file 7 — Source data Fig. 6 [file 44319_2025_496_MOESM7_ESM.zip › Figure 6/6B/20230821PDGFA_Fig/DTR_overlay.tif]

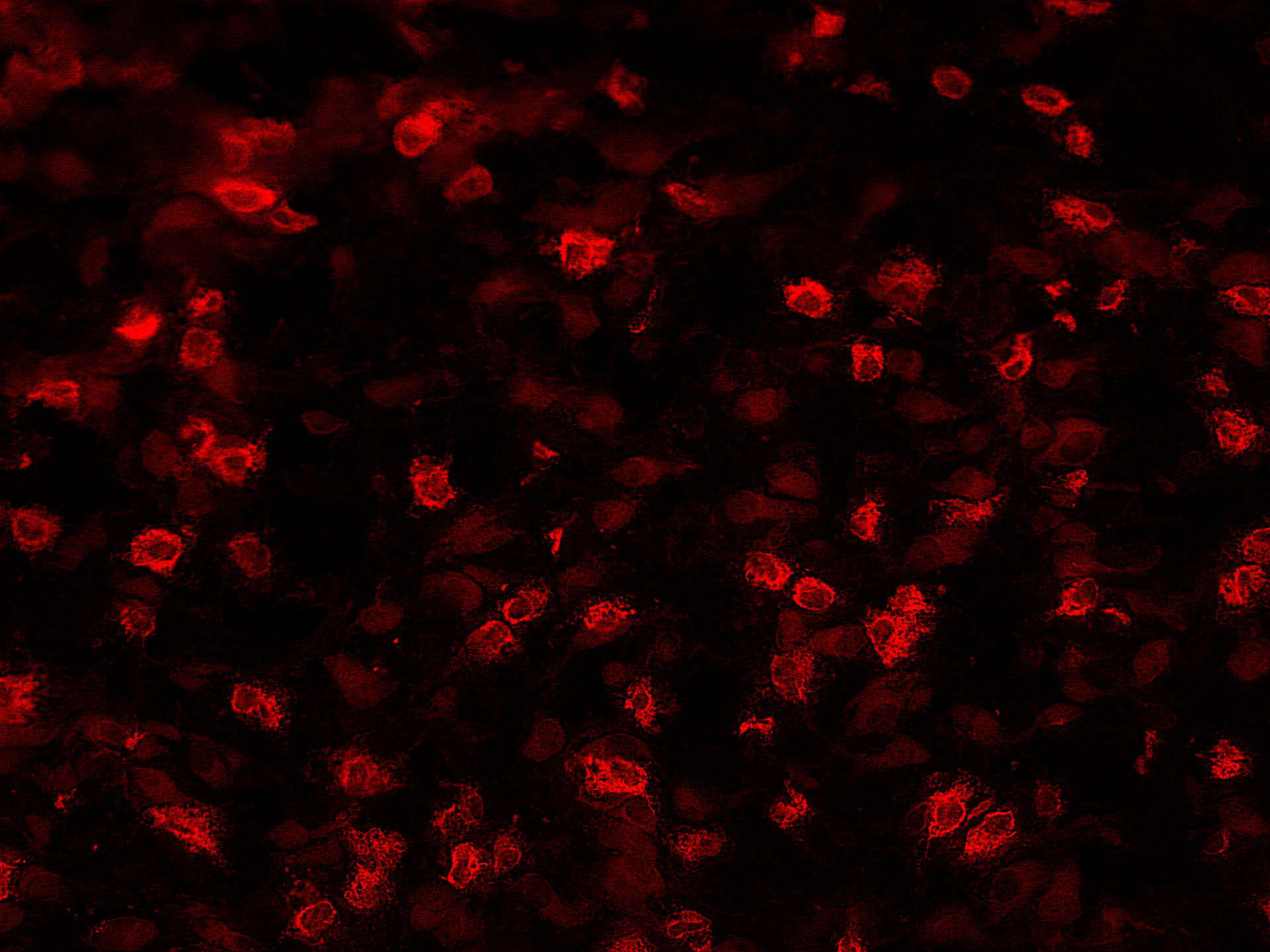

Supplement: Supplementary file 7 — Source data Fig. 6 [file 44319_2025_496_MOESM7_ESM.zip › Figure 6/6B/20230821PDGFA_Fig/DTR_PDGFA.tif]

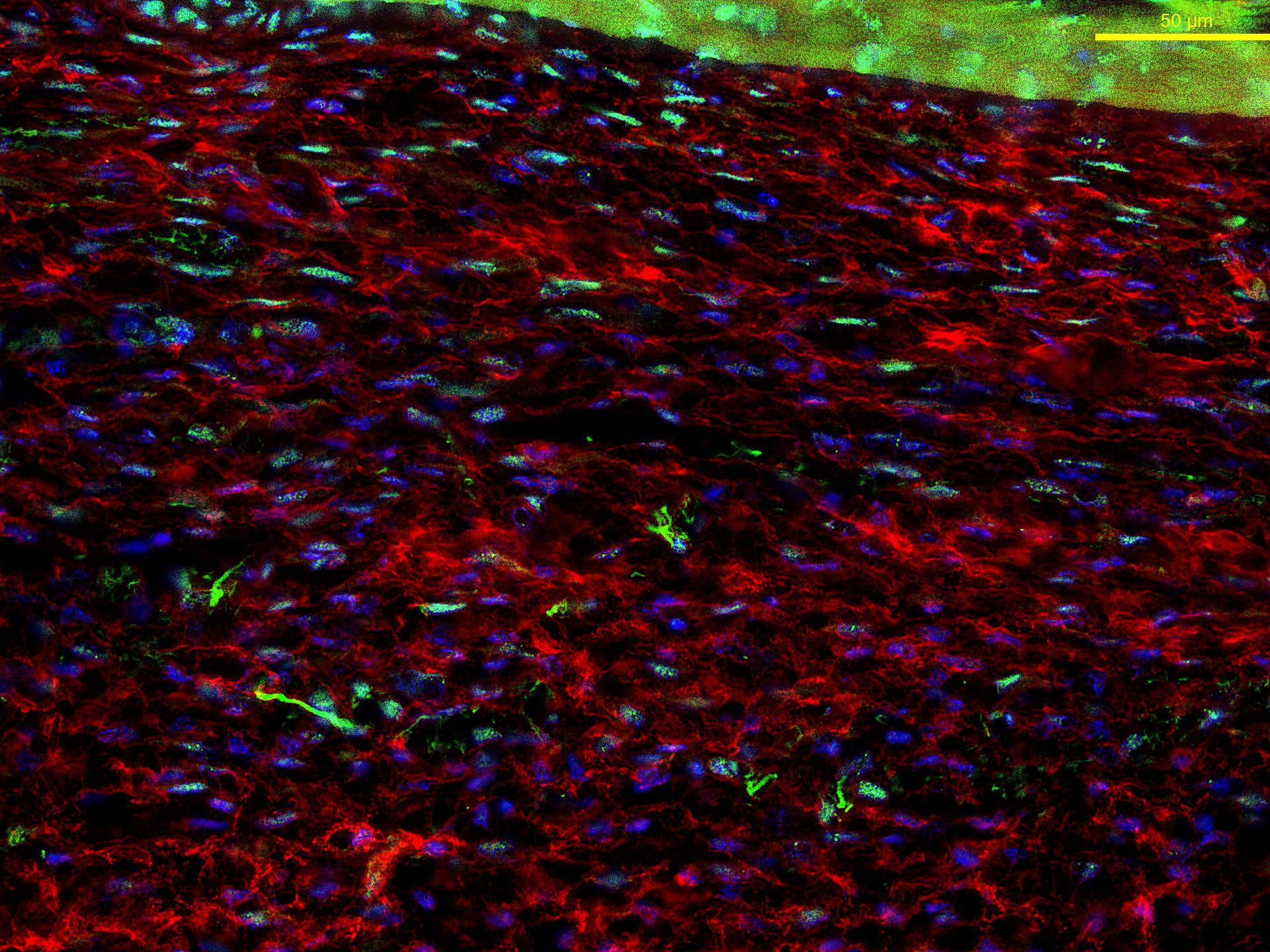

Supplement: Supplementary file 7 — Source data Fig. 6 [file 44319_2025_496_MOESM7_ESM.zip › Figure 6/6D/highmagnification.tif]

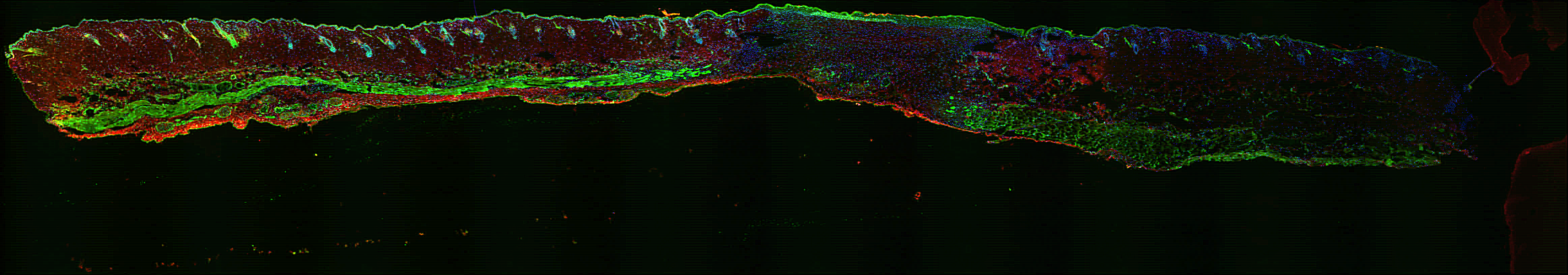

Supplement: Supplementary file 7 — Source data Fig. 6 [file 44319_2025_496_MOESM7_ESM.zip › Figure 6/6D/lowmagnification.tif]

## Slide 1
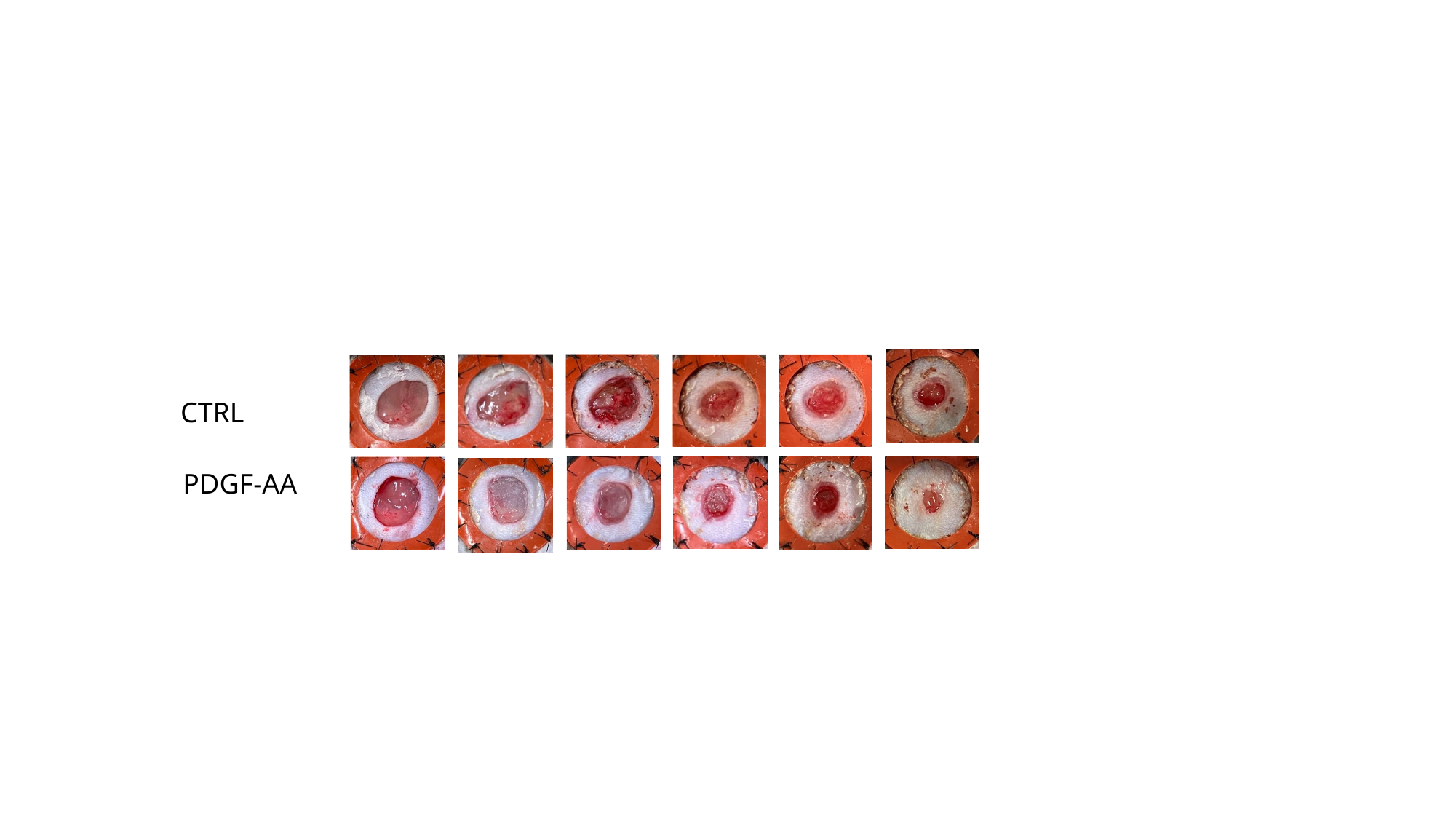

CTRL
PDGF-AA

Supplement: Supplementary file 7 — Source data Fig. 6 [file 44319_2025_496_MOESM7_ESM.zip › Figure 6/6G/PDGFA_wound.pptx]

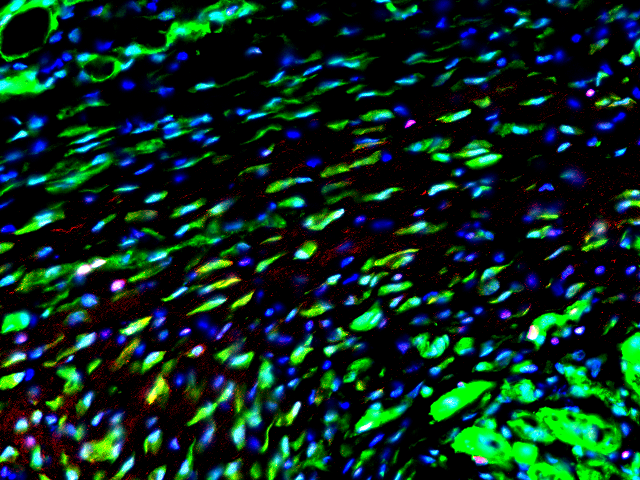

Supplement: Supplementary file 7 — Source data Fig. 6 [file 44319_2025_496_MOESM7_ESM.zip › Figure 6/6I/PBS.tif]

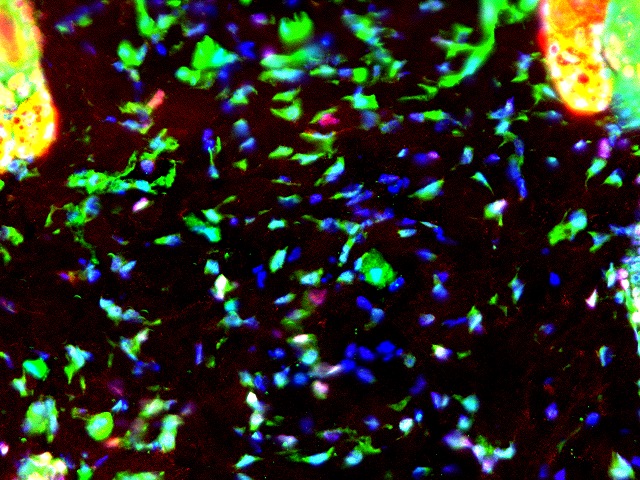

Supplement: Supplementary file 7 — Source data Fig. 6 [file 44319_2025_496_MOESM7_ESM.zip › Figure 6/6I/PDGF-AA.tif]
