# Supplementary material for: CD206+ macrophages facilitate wound healing through interactions with Gpnmbhi fibroblasts
Source: EMBO Rep. 2025 Jun 10;26(14):3679–704. doi: 10.1038/s44319-025-00496-4 (PMC12287335; doi:10.1038/s44319-025-00496-4)
Supplement: Supplementary file 4 — Data Set EV2 [file 44319_2025_496_MOESM4_ESM.docx]

**Datasets EV 2**

**Top gene lists of macrophage subclusters**

**Cluster 0 *C1qa*^+^**

Cd4, Fxyd2, Igf1, Gm47283, Lpl, Mid1, Ccl8, Aspn, Atp6v0d2, Arhgap19, Vcam1, Gdf15, Gas6, Tanc2, Aebp1, Igfbp4, Mgl2, Dcn, Serpinb6a, Sprr1b, Rapsn, Fcrls, Lum, Mmp12, Ppfibp2, Rarres2, Ifi202b, Serpina3n, Mmp2, Krt10, Col1a2, Mmp3, Tspan4, Ttyh2, Col1a1, Acss1, Blnk, Sprr1a, Serping1, Hpgds, Gdpd1, Spic, Epcam, Cpxm1, Anpep, Syngr1, Ctsk, Krt77, Serpinb5, Sparc, Serpinb2, Cd72, Apod, Krt1, Fxyd3, Clec4b1, Perp, Cxcl12, Klk7, Cst6, Col5a2, Krt5, C1qa, Ly6g6c, Serpinf1, Adam19, Cela1, Col3a1, Lypd3, S100a14, Gpx3, Il33, Pltp, Bgn, Dnmt3a, Calm4, Pparg, C1qc, Wwp1, Gramd1b, Itga9, Pmp22, S100a16, Zfp704, Timp2, Vkorc1, Krt16, Ptx3, Reps2, Selenop, Slamf9, Krt14, Plpp3, Folr2, Lpcat1, Krtdap, Slc29a1, Cpq, Serpinb9, Sgpp1, Fstl1, Tns1, Pdgfa, Slamf8, Ms4a7, 2310046K23Rik, Lgals7, Cgref1, Slamf7, Clec10a, Tmem26, Sbsn, Abca9, Npl, Htra1, Slc25a10, Tacstd2, Igfbp7, Cd63, C1qb, Fblim1, Cd81, Defb6, Stmn1, Pkp4, Gpt2, Nr1d2, Fam83f, Ly6d, Frmd4b, Tmem176a, Ahnak2, Krt17, Gpnmb, Fbxo32, Fabp4, Ly9, Ccdc34, BC100530, Hmgn1, Gm5547, Plet1, Arhgef12, Elmsan1, Slc30a1, Ebi3, Hvcn1, Fosb, Tmem176b, Jup, H2-Eb1, Itgax, Dnah2, Il11ra1, Gga2, Tmem140, Pdgfb, Pdlim4, Lipa, Krt79, Fnbp1l, Nrp1, H2-Aa, Traf5, Rassf3, Fem1b, Fcgrt, H2-Ab1, Pla2g15, Axl, Smagp, Tmem267, Il10, Slc27a1, Jmy, Tbc1d4, Hexb, Zmynd15, Epb41l4aos, Pld3, C4b, Clec12a, Mtss1, Trem2, Itsn1, Rcan1, Pxdc1, Mcoln2, Hebp1, Ckb, Ighm, Sfn, Mkl1, Cipc, Ndrg1, Rnase4, Plxnc1, Hist1h2bc, Slc11a2, Rgs1

**Cluster 1 *Vcan*^+^**

Wfdc21, Lcn2, Ifitm6, Hp, Sell, Vcan, Lrg1, Mcemp1, Mmp8, Jaml, Trem3, F5, Plcb1, Cd177, Prtn3, Gnb5, Rab44, Flt3, Il18rap, Ifitm1, Htr7, Tgm1, Slc16a3, Tarm1, Ltb4r1, Rdh12, Trem1, Gm15283, Smpdl3b, Il1r2, Gm9733, Casp6, Chil3, Hdc, Slpi, Plac8, Napsa, Treml4, Saa3, Rdh11, Gm26699, Il1b, Gm49368, Ugt1a6a, Dgat2, Crtc3, Pi16, Slc15a2, Dusp16, Igfbp6, Klra2, Nod2, Pbx1, Itgal, Pik3cg, Acsf2, Itga1, Cish, Sipa1l1, Dmkn, Sdf2l1, Slfn1, Inpp1, Sorl1, Slc4a11, Samsn1, Abcc4, Adap2os, Lpar6, Dgkg, Pnkp, Gm26669, Stn1, Arhgap26, Uck2, 1600014C10Rik, Spint1, Fmnl1, Lrrk2, Clec4e, Il1rap, Cmtr1, Cd80, Abcb1a, Snx14, Map3k14, Ifitm2, Gm26740, Gsr, Matn4, Creld2, Card9, Cxxc5, S100a11, Coro1a, Rap2a, Rbm10, Sqor, Eed, Adora2b, Lax1, Gda, Bin2, Gm48065, Zfp52, Svil, Gm5150, Ms4a4b, Ppp1r21, Piwil2, Tnfaip8l2, Gapt, Ifitm3, Id1, Tes, Ugt1a7c, Ass1, Nlrx1, Stat4, Maml3, Emb, Ttll3, Wrn, Cenpq, Glipr2, AI839979, Bri3bp, Magohb, Arg2, Mgst1, Slc35b1, Gpr141, Nucb2, Upp1, Ramp3, Hhex, Vdr, Trps1, Ceacam1, Dcun1d3, Tlr4, Hinfp, 3110082I17Rik, Cux1, Acvr2a, Ccdc84, Ccdc71l, Tmem39a, Lyrm4, Dna2, Ncf4, Abca7, C5ar2, Slc7a11, Fcgr3, Cenpl, Sesn2, Pkd1l2, Adora2a, I830077J02Rik, Ndufaf7, Filip1l, Smchd1, Prkce, D16Ertd472e, Orai1, C130050O18Rik, Lyrm1, Cdk2ap2, Ssr2, Pde3b, Gpr84, Havcr2, Cass4, Gk, Coq2, Gm35154, Tjap1, Ufl1, Nedd9, Sept6, Gale, Gm29994, Gm16133, Flot1, Sirpb1c, Dnajb11, Med7, Stap1, Lrp10, Ufsp2, Glis3, Creb3l2, Mapkapk3, Samhd1, Dpep2, Trabd, F10, Pmvk, Auh

**Cluster 2 *Ccl7*^+^**

Calml4, Ccl7, Cbr2, Acod1, Ltc4s, Tmem8, Spon1, Tfeb, Enpp1, Slc26a11, Ccl2, Siglec1, Stxbp6, Oasl1, Ccl6, Ly6c2, Epb41l1, Slc28a2, Rarg, Syne3, Snx30, Wwc1, Tppp3, Cdc14b, Gm13212, Man2b2, Rnf141, Dusp7, Kcnn4, Il10, Tmem104, Fcgrt, Hip1, Cipc, Ly6a, Gprc5a, Folr2, Mcoln2, Parp3, Rgl1, Gpt2, Nfic, Mfsd12, Hk3, Tob1, Gnpda1, P2ry6, Tpcn1, Cdc42ep4, Adcy7, Sh3bp5, P2ry2, Irf2bpl, Cfb, Oaf, Trp53bp2, Hk1, Tatdn2, Psd3, Cdc42ep2, Nt5dc2, Cebpa, Gramd3, Phldb1, Marcksl1, B3gnt8, Nr1h3, Dhrs3, Slc2a6, Sdc3, Itpr1, Tnfrsf1b, Pira2, Homer3, Lat2, Rell1, Dennd4b, Stx11, Gm28875, Kif13b, Fnip2, Lifr, Ptpn23, Rnf41, 2310022A10Rik, Pdxk, Dgkz, Rhob, Bahd1, Rars, Slc8b1, Ifi27l2a, Itga5, Arap1, Cryba4, Naaa, Cxcr4, Snx27, Coro1c, Ldlrap1, Bcl2l1, Serpinb8, Zcchc2, Il18bp, Cdt1, Piezo1, Lasp1, Ncf1, Eps8, Fbxw7, Ednrb, Kras, Ubtd1, Vat1, Rab3gap2, Dennd4c, Ern1, Dirc2, F13a1, Tspan14, 3110043O21Rik, Cln5, Pepd, Tulp4, Asap1, Ptafr, Ttc7, Ms4a8a, Fam214b, Lgals9, Plin3, Rassf2, Mrc1, Stom, St6galnac4, Zdhhc18, Mef2a, Parvb, Rhov, Atp6v1c1, B4galt1, Slc9a3r1, Tcp11l2, Mcfd2, Cd300lf, Ezr, Camkk2, Cflar, Peli1, Dtnbp1, Slc35e4, Abcg1, Ilf3, Plxna1, Slc17a5, Tmem184b, Capn2, Acp2, Pfkfb3, Amdhd2, Dcxr, Actn4, Rasgrp4, Lyst, Nceh1, Irak2, Kif1c, Ifi205, Gas7, Ldlr, Washc5, Lhfpl2, Il17ra, Fgr, Lpxn, Rab31, Cpne2, Pcyt1a, Map7d1, Homer1, Tbc1d2b, Slc29a3, Ncoa3, Tet3, Galnt6, Rasa3, Plekho1, Adgre5, Lcp2, Gba, Tbc1d9, Ckb, Pacs2, Lgals1, Slc43a2, Aoah, Rhbdf2, Hpcal1, Slc16a6, Impact

**Cluster 3 *Cd9*^+^**

Arg1, Cav1, AA467197, Nt5e, AC110211.1, Raet1e, Cxcl3, Ptges, Cd9, Ppbp, Fn1, Ccl24, Pdpn, Sema3c, Bdh2, Il1a, Ccl3, Fmnl2, Gnat3, Pcdh7, Tnfrsf9, Met, Dglucy, Socs2, Cav2, Fpr1, Slc7a2, Cd24a, Grina, Actn1, Sdc1, Spp1, Ptgfrn, Spry2, Tfec, Raet1d, Bnip3, Dmxl2, Tnfrsf23, Fam20a, Src, Rai14, Clec5a, 1700012B09Rik, Ptger2, Itga1, Carmil1, Igf2r, Papss2, Tnfrsf26, Cgnl1, Flrt2, Slc27a4, Inhba, Upp1, Ecm1, Tcf7l2, P2ry10b, Timp1, Dclre1b, Cd2, Abcc3, Arhgap24, Rffl, F7, Pgk1, Prnp, Myo1e, Pecam1, Chst11, Akap7, F10, Cxcl2, Ifitm1, Rdh12, Rnf128, Tlr4, Hopx, Mmp14, Ass1, Net1, Pdlim7, Dubr, Mtm1, Aldoa, Pla1a, Bach2, Adssl1, Pld1, Kcnk13, Ero1l, Pmepa1, Clec4d, Plp2, Cdh1, Aifm2, Dhrs9, Jag1, Fmnl3, Dtx4, Adgrl2, Fam162a, Pf4, A930007I19Rik, Slc7a8, Iqsec1, Ikbke, Lamc1, Txn1, Fmn1, Nfatc1, Cib2, Glrx, Grk5, Glis3, Prmt2, Pygl, Pde4d, Eno1, Mif, Sgms2, Zfp36l1, Clec4n, Rsbn1, Gpr68, Prdx6, Acot7, Tmem119, Rgcc, Mitf, Arg2, Mgst1, Fam20c, Cacna1d, Tlr13, 2510009E07Rik, Thbd, Slc7a11, Gpr84, Retreg1, Rab44, Slpi, Spint1, Gpat3, Mertk, Lpar1, Msr1, Maml3, Prdx5, Tes, Acsl1, Nek6, Pkd2, Rapgef1, Ccr1, Cry1, Olfml3, Slc37a2, Ctsl, Il7r, Dip2a, Gnptab, Col4a1, Plekhn1, Arrdc4, Itgam, Fpr2, Gapdh, Pkm, Pfkl, Tpi1, Bcl2a1b, Glipr2, Cpd, Atxn1, Srgn, Slc44a1, Abcc5, Cd80, Spire1, Plcb1, Agap1, Rdh11, Cd36, Adam8, Map3k20, Gsap, Sav1, Slc22a4, Thbs1, Gsr, Zeb2, Ptpn12, Deptor, Ppfibp1, Cd93, Il1rn, Hif1a, Pid1, Tapt1

**Cluster 4 *Klf2*^+^**

AL732506.1, Hspa1a, Gm26759, Ccnb1ip1, Engase, Klf2, Hspa1b, Hist4h4, Bmyc, Hspb1, Ciart, Ankrd37, 1110035H17Rik, Fam71a, Pald1, Nr4a1, Il16, Wee1, Slc5a3, Grasp, Hes1, Fam69a, Spred3, Psme2b, Egr1, 5430416N02Rik, Fosb, Atf3, Cx3cr1, Dusp8, Gpr34, 1810011H11Rik, Id3, Zbtb10, 2700038G22Rik, Flt3l, Txnip, Bmf, Batf3, Rasgef1b, Btg2, Wdr12, Tyw1, Dtd2, Ciita, Lars2, Cd79b, 9930111J21Rik2, Ppfia4, Tnfsf9, Rcsd1, Sh2d1b1, Tbl3, Bbc3, Nup85, Ormdl3, Narf, Gm26917, Zkscan14, Gm26890, Slc20a1, Trim5, Dhfr, Hcar2, Msh3, Gdpd5, Urb1, Fos, Bcl2, Nudt22, Relt, Zfp715, Rassf5, Chd3os, Fam53b, Prr3, Zfp422, Cdpf1, Zfand1, Rbfa, Per1, Nudcd1, Mb21d1, Brf2, Snhg12, Ccrl2, Fgd2, Car9, Tmcc3, Trim47, Pim3, Usp2, Nudt13, Tifab, Trp53rkb, Dnajb1, Rgs2, Ccl4, Abcg3, Kptn, Chka, Pmaip1, Qtrt1, Card9, Pdcd2, Mettl2, Tagap, Plcxd2, Sh3bp1, Lyl1, Dcakd, Atp13a2, Zfp397, Lmo2, Slc12a9, Ssh2, Usp36, Cdkn2d, Cbx4, Zfp395, Aim2, Adrb2, Id1, 1810043G02Rik, Tmem229b, Usf1, Klf4, Rasgrp1, Ifi44, Fgd4, Kcnk6, Pdlim2, Dusp2, Slc52a2, Smagp, Dclre1c, Thap11, Sesn1, Gm15987, Arhgap39, B4galt3, Hsp90aa1, Stk38l, Sac3d1, Snta1, Irf1, Abi3, Adap1, Myo7a, Dusp1, Limd2, Sdhaf1, Map3k8, Gm26740, Rps6ka1, Gadd45b, Tpgs1, Nfkbid, Fam26f, Ccnd2, Slc7a6os, Sdad1, Nr1d1, Crebrf, Mknk1, Themis2, Hsph1, Jun, Apobec3, Kdr, Slamf9, Swap70, Itga6, Hspe1, Rnd3, Sirt3, Slc25a25, Clmp, Xpa, Manba, P2ry12, Mphosph10, Ldb1, Sap30, Rfng, Ifit2, Cd72, Mvb12b, Jund, Plk2, Fads1, A630001G21Rik, Gdf15, Bphl, Dedd2, Ehmt2, Arl4d, Prkra, Fgfr1op, Frat2

**Cluster 5 *Ifit1*^+^**

Ifit1bl1, Ifit3b, Ifit3, Ifit2, Apol9b, BC147527, Gm4951, Cxcl10, Cmpk2, Tnfsf10, Ifi208, Ifit1, Gbp5, Iigp1, Ifi214, Ddx4, Rsad2, Serpina3g, Gm5431, Tlr3, Phf11c, Mx1, Ifi206, Ifi47, 9930111J21Rik2, Slfn9, Herc6, Timeless, Cxcl9, Usp18, Ccr7, A530040E14Rik, Phf11b, Isg20, Gbp2, Phf11a, Ccnd1, Ifi213, Phf11d, Psme2b, Ccl5, Themis2, Fap, Igtp, Ccnd2, Ifi44, Klrk1, Nt5c3, Fam26f, Irgm2, Sema7a, Pml, Gbp4, Rilpl1, Il27, Ddx60, Trim14, Irgm1, Trim30c, Mitd1, Isg15, Setdb2, Rtn1, Batf2, Gbp8, Znfx1, Gbp3, Il15, Ttll9, Sass6, Daxx, Tor3a, Stat2, Ifi209, Ifih1, Oasl1, P2ry14, Nectin4, Asb13, Ctnnd2, Gbp7, 2310031A07Rik, Mov10, AW112010, Ddx58, Klrd1, Mmp13, Slfn5, Parp9, Parp14, Cd209a, Irf7, Ube2l6, Dtx3l, Cd40, C130026I21Rik, Parp10, BC051226, Uaca, Aldh1b1, Samd9l, Slc25a22, Apobec3, Mndal, Trim21, Trim30b, Trim30d, Gnb4, Gm8369, A530032D15Rik, Ifi205, I830077J02Rik, Tpst1, Sp140, Klrb1b, Sco1, Ppm1k, Ifi211, Mlkl, Oas3, Hmgn3, Il15ra, Treml2, P2ry10, Parp12, Frmd4a, Mb21d1, Ncoa7, Xaf1, Lipg, Rbm43, AW011738, Slfn8, Nlrc5, Nmi, Slfn1, Rin2, Pttg1, H2-T22, Zbp1, Zc3h7b, Rtp4, Ifi203, Kmo, Pnp, Rhof, Tap1, Kdr, Oasl2, Slfn4, Stat1, Oas2, Zufsp, Ciita, Trim30a, Slamf9, Cnp, Trafd1, Slamf8, Ifi35, Ms4a4b, Serpinb9, Eif2ak2, Ikzf1, Ccl12, March5, Dhx58, Axl, Rubcnl, Mthfr, Helz2, Ifi204, Tpx2, Tdrd7, Scimp, Sp110, Tmem140, Fam241a, Casp3, Nmral1, Gbp9, Epsti1, 1600014C10Rik, Fam46a, Ms4a4c, Sgcb, Rnf213, Gpsm2, AA467197, Pnpt1, Trim12c, Sap30, Slco3a1, Irf1, Uba7, Psmb9, Amigo3, Sp100, Tlr8, Tmem209
