# Supplementary material for: CD206+ macrophages facilitate wound healing through interactions with Gpnmbhi fibroblasts
Source: EMBO Rep. 2025 Jun 10;26(14):3679–704. doi: 10.1038/s44319-025-00496-4 (PMC12287335; doi:10.1038/s44319-025-00496-4)
Supplement: Supplementary file 8 — Expanded View Figures [file 44319_2025_496_MOESM8_ESM.pdf]

Expanded View Figures

Figure EV1. Flow cytometric analysis of wound tissue.

Wound tissues were harvested from C57BL/6 mice at the indicated times after injury and analyzed by flow cytometry. (**A**) Gating strategy. Live cells were sub-gated based on CD45 and CD11b expression, and $CD45^{+}CD11b^{+}$ myeloid cells were sub-gated based on the expression of Ly6G. $Ly6G^{-}$ cells were further sub-gated based on CD206 and F4/80 expression to reveal the $CD206^{+}F4/80^{+}$ population. $CD45^{-}CD11b^{-}$ cells were sub-gated based on CD31 and Epcam expression. $CD31^{-}Epcam^{-}CD26^{+}$ cells were defined as fibroblasts. The diagram for the control mice in Fig. 1B is presented again as a CD206 and F4/80 gating plot to show the correspondence. (**B**) Isotype control of the wound macrophages. The isotype control for wound macrophages was used to assess CD206 and F4/80 expression. The cells were stained with the respective antibodies, and instrument settings and compensation were adjusted using the corresponding isotype control. The analysis was performed using FlowJo software ($n = 3$ wounds, biological replicates). The diagram for the control mice in Fig. 1B is presented again to show the correspondence. (**C**) Fluorescent minus one (FMO) control for the CD206 and F4/80 staining to identify and gate cells in flow cytometry experiments ($n = 3$ wounds, biological replicates). (**D–F**) Temporal changes in the numbers of fibroblasts and macrophages in wounds. The number of $CD206^{+}$ macrophages per wound over time is shown (**D**). Data are shown as means ± SD. *$p = 0.0008$ (day 3), 0.0002 (day 5), 0.0685 (day 7), 0.9886 (day 14) by one-way ANOVA followed by Dunnett's multiple comparisons test ($n = 3$ mice/group, biological replicates). The number of $CD26^{+}$ fibroblasts at 5 days post injury (**E**) and the proportion of $CD45^{+}$ cells relative to the total number of live cells (**F**) in our mouse skin injury model are shown. Data are shown as means ± SD. *$P = 0.0285$ (**E**), 0.0493 (**F**) by unpaired two-tailed Student's t test ($n = 3$ mice/group, biological replicates).

 © The Author(s)

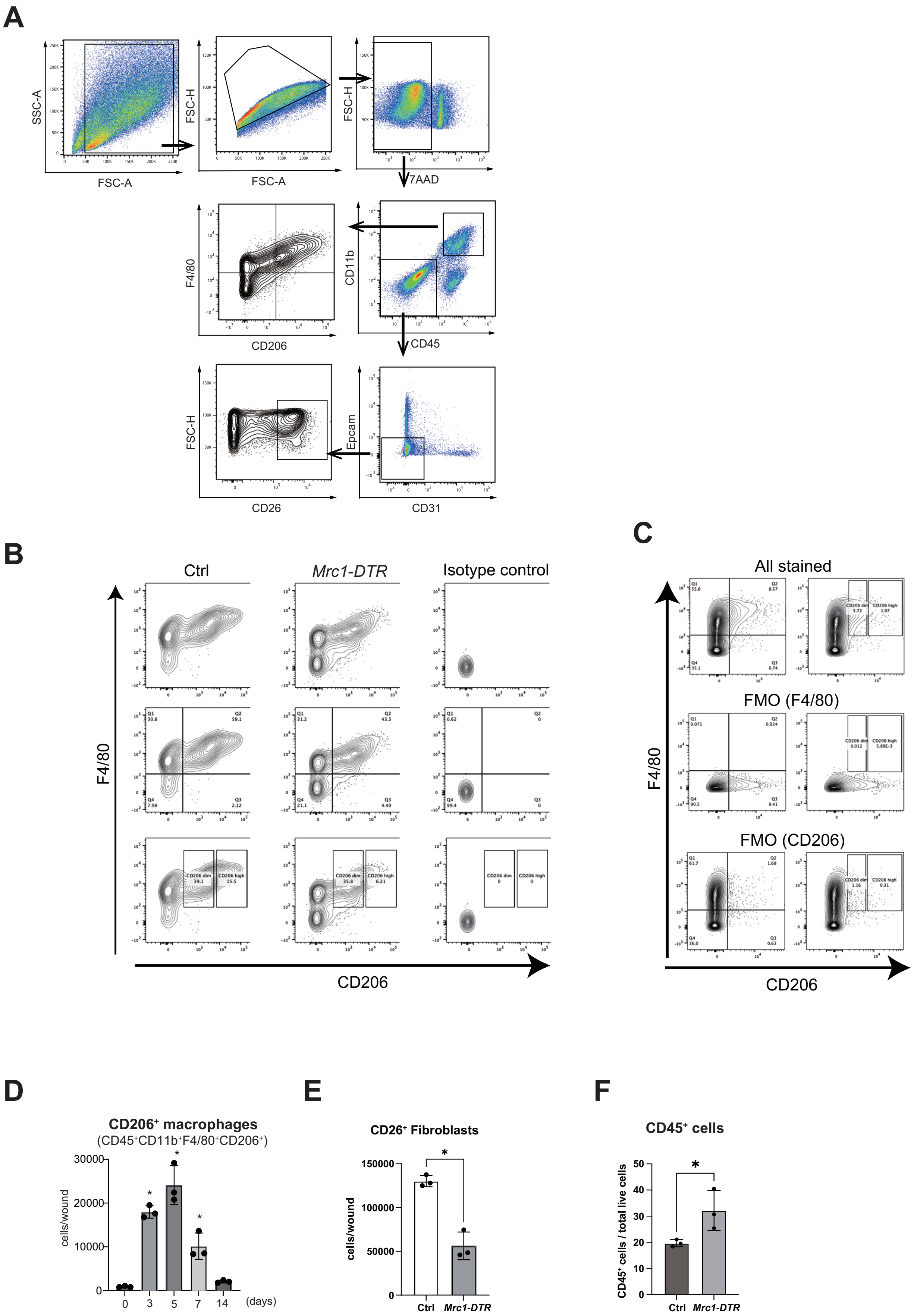
A
SSC-A
FSC-A
FSC-H
FSC-A
FSC-H
7AAD
F4/80
CD206
CD11b
CD45
FSC-H
CD26
Epcam
CD31
B
Ctrl
Mrc1-DTR
Isotype control
F4/80
CD206
C
All stained
FMO (F4/80)
FMO (CD206)
F4/80
CD206
D
CD206⁺ macrophages
(CD45⁺CD11b⁺F4/80⁺CD206⁺)
cells/wound
(days)
E
CD26⁺ Fibroblasts
cells/wound
Ctrl
Mrc1-DTR
F
CD45⁺ cells
CD45⁺ cells / total live cells
Ctrl
Mrc1-DTR

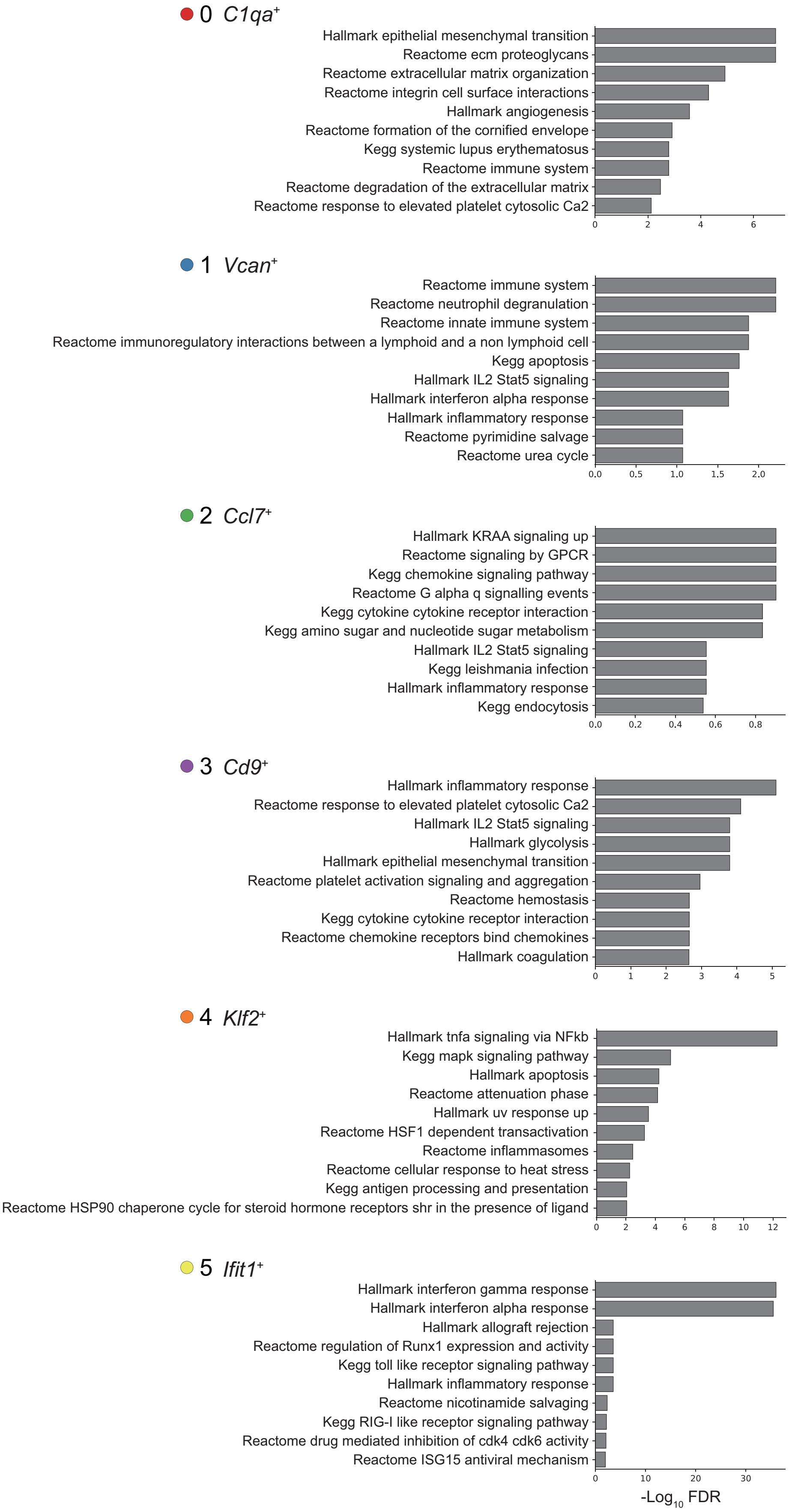
0 C1qa+
Hallmark epithelial mesenchymal transition
Reactome ecm proteoglycans
Reactome extracellular matrix organization
Reactome integrin cell surface interactions
Hallmark angiogenesis
Reactome formation of the cornified envelope
Kegg systemic lupus erythematosus
Reactome immune system
Reactome degradation of the extracellular matrix
Reactome response to elevated platelet cytosolic Ca2
0 2 4 6
1 Vcan+
Reactome immune system
Reactome neutrophil degranulation
Reactome innate immune system
Reactome immunoregulatory interactions between a lymphoid and a non lymphoid cell
Kegg apoptosis
Hallmark IL2 Stat5 signaling
Hallmark interferon alpha response
Hallmark inflammatory response
Reactome pyrimidine salvage
Reactome urea cycle
0.0 0.5 1.0 1.5 2.0
2 Ccl7+
Hallmark KRAA signaling up
Reactome signaling by GPCR
Kegg chemokine signaling pathway
Reactome G alpha q signalling events
Kegg cytokine cytokine receptor interaction
Kegg amino sugar and nucleotide sugar metabolism
Hallmark IL2 Stat5 signaling
Kegg leishmania infection
Hallmark inflammatory response
Kegg endocytosis
0.0 0.2 0.4 0.6 0.8
3 Cd9+
Hallmark inflammatory response
Reactome response to elevated platelet cytosolic Ca2
Hallmark IL2 Stat5 signaling
Hallmark glycolysis
Hallmark epithelial mesenchymal transition
Reactome platelet activation signaling and aggregation
Reactome hemostasis
Kegg cytokine cytokine receptor interaction
Reactome chemokine receptors bind chemokines
Hallmark coagulation
0 1 2 3 4 5
4 Klf2+
Hallmark tnfa signaling via NFkb
Kegg mapk signaling pathway
Hallmark apoptosis
Reactome attenuation phase
Hallmark uv response up
Reactome HSF1 dependent transactivation
Reactome inflammasomes
Reactome cellular response to heat stress
Kegg antigen processing and presentation
Reactome HSP90 chaperone cycle for steroid hormone receptors shr in the presence of ligand
0 2 4 6 8 10 12
5 Ifit1+
Hallmark interferon gamma response
Hallmark interferon alpha response
Hallmark allograft rejection
Reactome regulation of Runx1 expression and activity
Kegg toll like receptor signaling pathway
Hallmark inflammatory response
Reactome nicotinamide salvaging
Kegg RIG-I like receptor signaling pathway
Reactome drug mediated inhibition of cdk4 cdk6 activity
Reactome ISG15 antiviral mechanism
0 10 20 30
-Log10 FDR

© The Author(s)

Figure EV2. Gene sets enriched in highly expressed genes in macrophage subpopulations.

Over-representation analysis of the top 100 upregulated genes in each subpopulation, compared to the rest, with MSigDB hallmark, Reactome, and Kegg gene sets using decoupleR (Badia et al, 2022; Castanza et al, 2023; Subramanian et al, 2005). The top 10 terms are shown. A list of genes used in the analysis is presented in Dataset EV2.

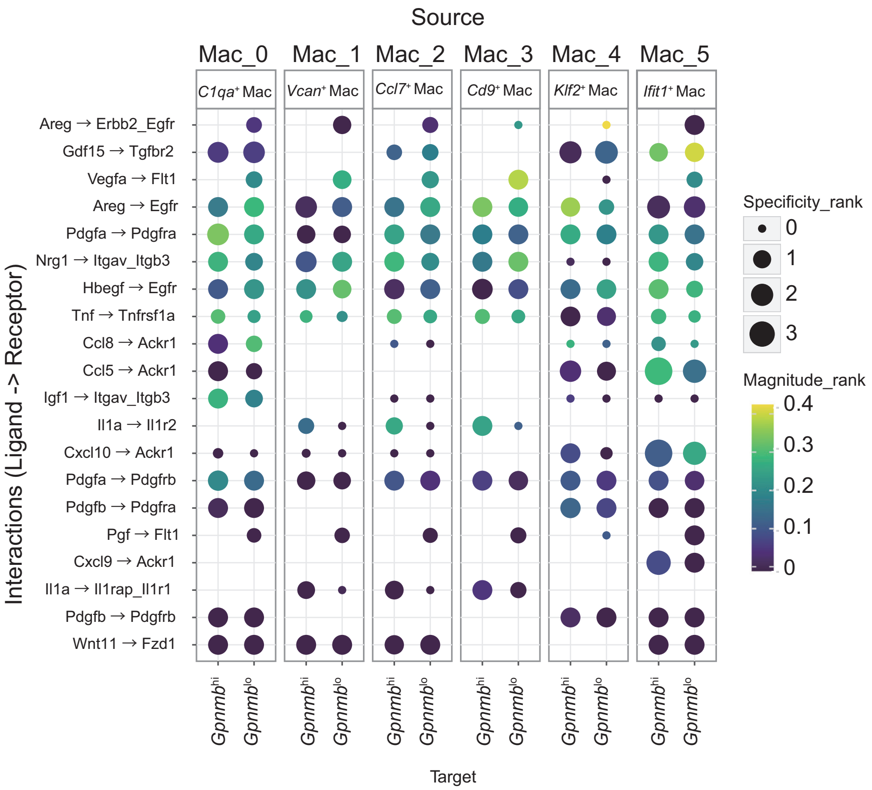

Figure EV3. Dot plot of ligand-receptor-interactions between macrophage subpopulations and *Gpnmb*^{hi}/*Gpnmb*^{lo} fibroblasts.

Top 20 ligand-receptor pairs of the aggregate consensus rank are shown. Macrophage subpopulations expressing ligands and fibroblast subpopulations expressing receptors are shown on the *x* axis. Ligand and cognate receptor combinations are shown on the *y* axis. Circle size denotes specificity rank, a measure of how specific an interaction is to a given pair of cell groups; color denotes magnitude rank showing the strength of ligand-receptor interaction.

 © The Author(s)

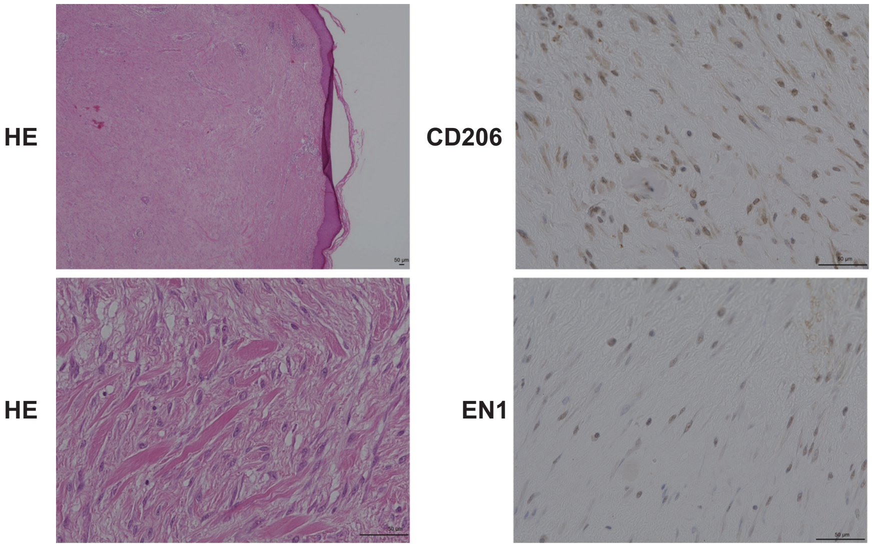

Figure EV4. Presence of CD206$^+$ macrophages and EN1$^+$ fibroblasts in human keloid tissue.

Representative histologic images of human keloid tissue stained with hematoxylin and eosin (HE) and immunostained for CD206 and EN1. Scale bars: 50 µm.

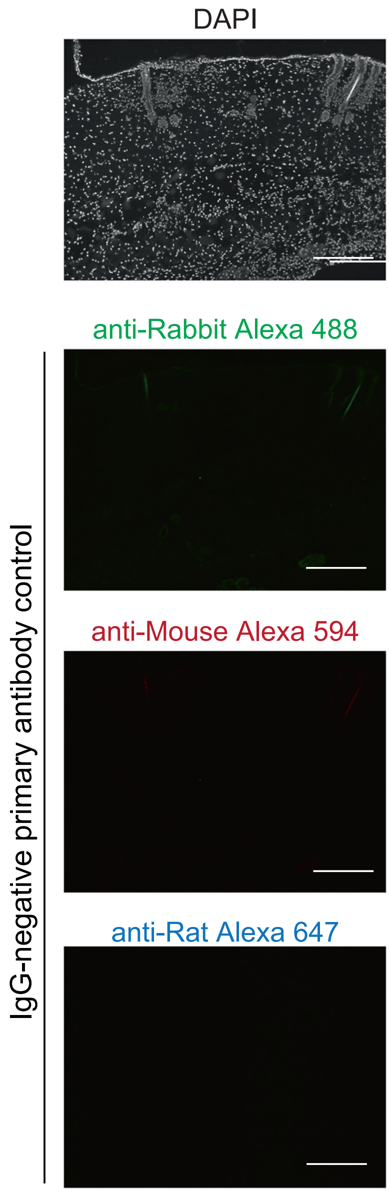

Figure EV5. Negative control for immunofluorescence staining for Fig. 5C,G.

Tissue sections were stained with secondary antibodies only to confirm the absence of non-specific fluorescence signals. Images show DAPI (white), Alexa Fluor 488 anti-Rabbit (green), Alexa Fluor 594 anti-Mouse (red), and Alexa Fluor 647 anti-Rat (blue). Scale bar: 200 μm.

 © The Author(s)
